# XPO7 is a tumor suppressor regulating p21^CIP1^-dependent senescence

**DOI:** 10.1101/gad.343269.120

**Published:** 2021-03-01

**Authors:** Andrew J. Innes, Bin Sun, Verena Wagner, Sharon Brookes, Domhnall McHugh, Joaquim Pombo, Rosa María Porreca, Gopuraja Dharmalingam, Santiago Vernia, Johannes Zuber, Jean-Baptiste Vannier, Ramón García-Escudero, Jesús Gil

**Affiliations:** 1 MRC London Institute of Medical Sciences (LMS), London W12 0NN, United Kingdom;; 2 Institute of Clinical Sciences (ICS), Faculty of Medicine, Imperial College London, London W12 0NN, United Kingdom;; 3 Centre for Haematology, Department of Immunology and Inflammation, Imperial College London, London W12 0NN, United Kingdom;; 4 Research Institute of Molecular Pathology (IMP), 1030 Vienna, Austria;; 5 Molecular Oncology Unit, Centro de Investigaciones Energéticas, Medioambientales y Tecnológicas (CIEMAT), 28040 Madrid, Spain;; 6 Research Institute 12 de Octubre (i+12), 28041 Madrid, Spain;; 7 Centro de Investigación Biomédica en Red de Cáncer (CIBERONC), 28029 Madrid, Spain

**Keywords:** XPO7, functional screen, senescence, tumor suppressor, TCF3, p21^CIP1^

## Abstract

Senescence is a key barrier to neoplastic transformation. To identify senescence regulators relevant to cancer, we screened a genome-wide shRNA library. Here, we describe exportin 7 (XPO7) as a novel regulator of senescence and validate its function in telomere-induced, replicative, and oncogene-induced senescence (OIS). XPO7 is a bidirectional transporter that regulates the nuclear-cytoplasmic shuttling of a broad range of substrates. Depletion of XPO7 results in reduced levels of TCF3 and an impaired induction of the cyclin-dependent kinase inhibitor p21^CIP1^ during OIS. Deletion of *XPO7* correlates with poorer overall survival in several cancer types. Moreover, depletion of XPO7 alleviated OIS and increased tumor formation in a mouse model of liver cancer. Our results suggest that XPO7 is a novel tumor suppressor that regulates p21^CIP1^ expression to control senescence and tumorigenesis.

## Abstract (precis)

In this study, Innes et al. sought to identify novel senescence regulators relevant to cancer. The authors screened a genome-wide shRNA library and identified exportin 7 (XPO7) as a novel regulator of senescence and validated its function in telomere-induced, replicative, and oncogene-induced senescence.

Senescence is a stress response that limits the replication of old, damaged, or preneoplastic cells. Senescence is defined by a stable cell cycle arrest and is associated with characteristic changes in transcription, metabolism, and chromatin organization (Gorgoulis et al. 2019). Senescent cells also produce a bioactive secretome known as the senescence-associated secretory phenotype (SASP) (Kuilman and Peeper 2009). Senescence occurs in response to diverse stresses such as oncogenic activation, irradiation, chemotherapeutic drugs, or telomere dysfunction (Herranz and Gil 2018). Replicative senescence of primary human fibroblasts is a stochastic process in part triggered by telomere attrition (Harley et al. 1990). In normal cells, telomeres are protected by the binding of the multiprotein Shelterin complex (de Lange 2005), which limits the accumulation of DNA damage at telomeres (Denchi and de Lange 2007). When telomeres erode, decreased Shelterin binding results in the accumulation of DNA damage and the induction of an unrestrained DNA damage response (DDR) (d'Adda di Fagagna et al. 2003). A DDR triggered by DNA hyperreplication is also observed during oncogene-induced senescence (OIS) (Bartkova et al. 2006; Di Micco et al. 2006). During OIS, DNA damage accumulates at fragile sites, including telomeres (Fumagalli et al. 2012; Suram et al. 2012). Telomeric damage and the consequent DDR is central to induction of senescence by different stressors (Victorelli and Passos 2017). However, other studies have also made the case for the contribution of telomere-independent mechanisms, such as the production of reactive oxygen species (Takahashi et al. 2006; Moiseeva et al. 2009; Correia-Melo et al. 2016).

As a result of DDR induction, p53 up-regulates the transcription of p21^CIP1^, a cyclin-dependent kinase inhibitor (CDKI). p16^INK4a^, another CDKI is also up-regulated during senescence and, together with p21^CIP1^, contributes to arrest of senescent cells. Collectively, the activation of the p53/p21^CIP1^ and p16^INK4a^/Rb tumor suppressor pathways underlies the implementation of the senescence growth arrest. Importantly, components of these pathways are among the most frequently altered genes in cancer, underlining the functional relationship between senescence and cancer. OIS occurs in premalignant lesions and limits tumorigenesis by arresting preneoplastic cells (Collado et al. 2005) and activating SASP-dependent immune surveillance (Kang et al. 2011). Consequently, advanced tumors bear mutations that allow them to avoid senescence, and senescence escape is recognized as a cancer hallmark (Hanahan and Weinberg 2011).

Genetic screens are powerful tools that have served to identify multiple regulators of senescence (Jacobs et al. 2000; Rowland et al. 2005; Acosta et al. 2008; Tordella et al. 2016; Wang et al. 2016). Given the intricate relationship between senescence and tumor suppression, these screens have also resulted in the identification of genes with important roles in cancer. In this study, we have screened for shRNAs blunting senescence induction. To this end, we have taken advantage of a dominant negative mutant of the shelterin component TRF2 to cause telomere dysfunction and trigger senescence (Jacobs and de Lange 2004). In the screen, we identified exportin 7 (XPO7) as a novel regulator of senescence. Given that XPO7 is often deleted or mutated in cancer, our results suggest that XPO7 is a novel tumor suppressor regulating senescence.

## Results

### TRF2^ΔBΔM^ expression as a model to induce senescence

**Figure 1.**
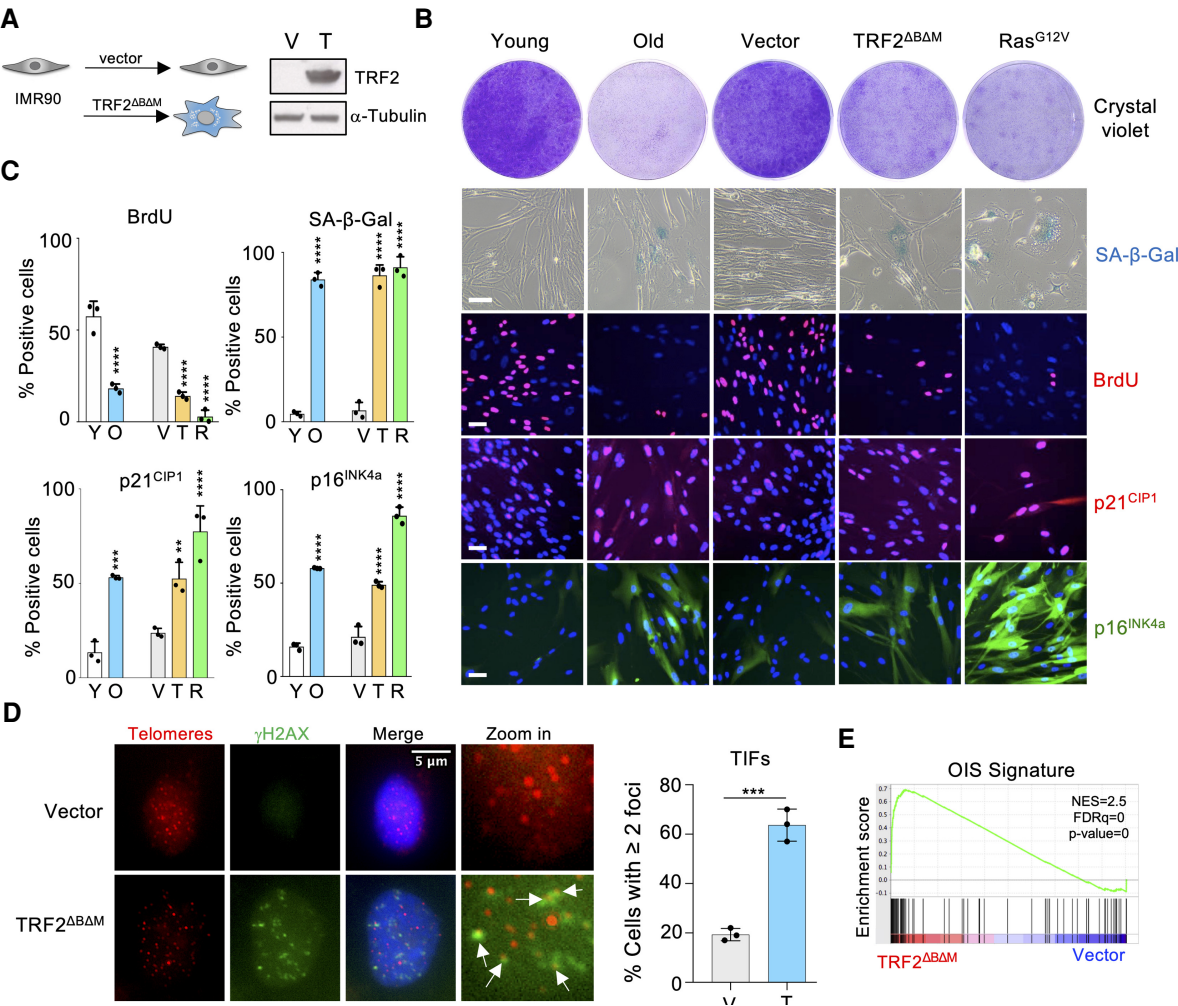
Expression of TRF2^ΔBΔM^ as a model of senescence caused by telomere dysfunction (*A*) Western blot confirming ectopic expression of TRF2^ΔBΔM^ in IMR90 cells. (V) Vector, (T) TRF2^ΔBΔM^. (*B*) Representative images of colony formation assay (crystal violet), SA-β-Gal staining, and BrdU, p21, and p16 immunofluorescence of proliferating (young) and senescent (old) IMR90, as well as IMR90 stably infected with TRF2^ΔBΔM^, H-Ras^G12V^, or empty vector. Scale bar, 50 µm. (*C*) Quantification of senescence markers shown in *B* (*n* = 3 for all experiments). (**)*P* < 0.01, (***) *P* < 0.001, (****) *P* < 0.0001 by one-way ANOVA with multiple comparison with respective control. (*D*) Representative images of immunofluorescence showing localization of γH2AX (green) and telomeres (red) in IMR90 cells infected with empty vector or TRF2^ΔBΔM^. Colocalizing signals (TIFs) are indicated with white arrows. Scale bar, 5 µm. Data are shown as percentage of cells with two or more colocalizing foci and are represented as mean ± SEM from three independent experiments. Statistical significance was calculated using a two-tailed Student's *t*-test. (***) *P* < 0.001. (*E*) GSEA showing the enrichment of a signature associated with OIS in the gene expression profile of IMR90 TRF2^ΔBΔM^ versus IMR90 vector. (NES) Normalized enriched score, (FDR) false discovery rate.

Replicative senescence of primary human fibroblasts is a stochastic process in part triggered by telomere attrition (Harley et al. 1990). The lengthy timeframe involved in senescence establishment makes studying replicative senescence of human fibroblasts challenging. Replicative senescence can be modeled by inducing telomere dysfunction (Jacobs and de Lange 2004). To this end, we infected IMR90 human fibroblasts with a vector expressing a dominant negative allele of the telomere binding protein TRF2 (TRF2^ΔBΔM^) (Fig. 1A) and compared the effect of TRF2^ΔBΔM^ expression with induction of replicative or oncogene-induced senescence in IMR90 cells (Fig. 1). Similar to replicative or oncogene-induced senescence in IMR90 cells, TRF2^ΔBΔM^ expression caused growth arrest, concomitant with the induction of the cyclin-dependent kinase inhibitors p16^INK4a^ and p21^CIP1^, and the induction of senescence-associated β-galactosidase activity (SA-β-Gal) (Fig. 1B,C). Expression of TRF2^ΔBΔM^ also resulted in increased DNA damage (Supplemental Fig. S1a) that accumulates at telomeres (Fig. 1D). To further characterize the response observed upon TRF2^ΔBΔM^ expression, we conducted transcriptomic analysis. Using gene set enrichment analysis (GSEA), we observed that signatures related to senescence and aging were enriched in IMR90 cells expressing TRF2^ΔBΔM^ (Fig. 1E; Supplemental Fig. S1b). Further GSEA shows that TRF2^ΔBΔM^ expression also caused the up-regulation of p53-target genes and a cell cycle arrest (Supplemental Fig. S1c). Overall, these data validate the use of TRF2^ΔBΔM^ expression as a model to study senescence.

### An shRNA screen identifies XPO7 as a regulator of senescence

**Figure 2.**
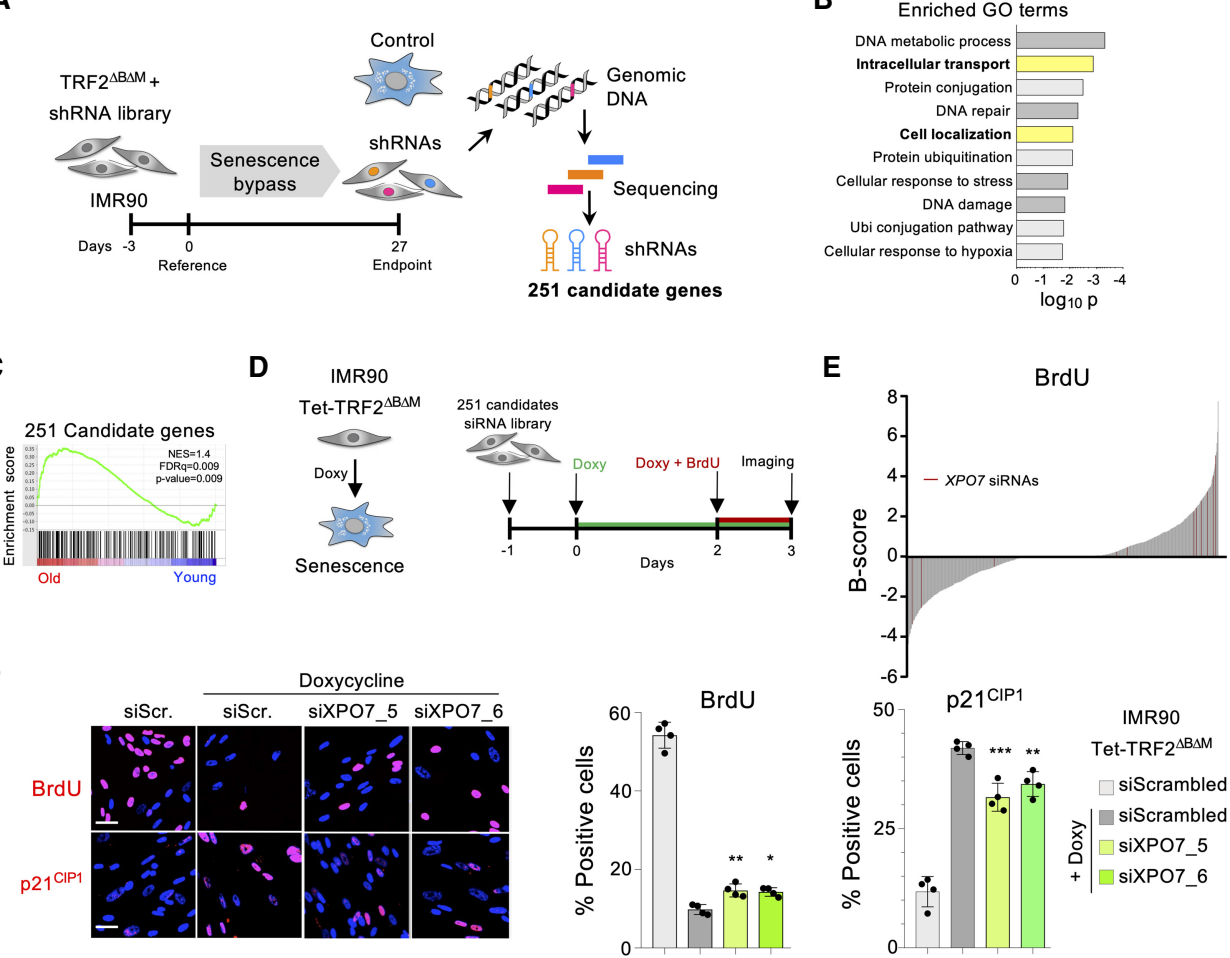
A genome-wide shRNA screen identifies XPO7 as a regulator of senescence. (*A*) Schematic model of the primary screen workflow. IMR90 fibroblasts were infected with a TRF2^ΔBΔM^ expression vector followed by a pooled genome-wide shRNA library. Samples for analysis of shRNA library representation were taken at the indicated times. The screen was performed in triplicate. After analysis, 251 genes were selected as candidates for a secondary screen. (*B*) Gene ontology (GO) analysis showing terms enriched in the list of 251 genes selected in the primary screen. (*C*) GSEA showing the enrichment of a signature comprising the 251 candidate genes in the gene expression profile of old IMR90 versus young IMR90 cells. (*D*) Schematic model of the secondary screen workflow. The screen was performed using IMR90 Tet-TRF2^ΔBΔM^ cells. Cells were reverse-transfected in 96-well plates with a custom RNAi library targeting the candidates selected in the primary screen. TRF2^ΔBΔM^ expression was induced by doxycycline addition. BrdU incorporation at day 3 after induction served as the surrogate readout of senescence bypass in the screen. Screen was performed in triplicate using four siRNAs targeting each gene. (*E*) B-score distribution of the BrdU incorporation in the secondary screen. Each line represents a replicate for a siRNA. siRNAs targeting XPO7 are shown in red. (*F*) IMR90:TRF2^ΔBΔM^ cells were reverse-transfected with siRNA targeting *XPO7*. Representative images (*left*) and quantification of BrdU incorporation (*middle*), and p21^CIP1^ immunofluorescence (*left*) are shown. Scale bar, 50 µm. (*n* = 4 biological replicates). (***) *P* < 0.001, (**) *P* < 0.01, (*) *P* < 0.05 by one-way ANOVA with multiple comparison with siScrambled + doxycycline cells. All error bars represent mean ± SD.

To identify novel regulators of senescence, we screened an shRNA library comprised of ∼58,000 shRNAs (Fig. 2A). IMR90 fibroblasts were transduced with a retroviral vector expressing TRF2^ΔBΔM^ followed by lentiviral transduction with the shRNA library. Cells were passaged to enrich for shRNAs that avoided the senescence-mediated growth arrest. Enriched shRNAs were identified, taking advantage of next-generation sequencing. Two-hundred-fifty-one candidate genes were selected (Fig. 2A). We conducted gene ontology (GO) analysis using the list of candidate genes and observed an enrichment of processes such as DNA damage or DNA repair (Fig. 2B) that have been previously implicated with the establishment of senescence (d'Adda di Fagagna 2008). Interestingly, other processes enriched among the candidate genes were intracellular transport and cellular localization (Fig. 2B; Supplemental Fig. S2a). Moreover, using GSEA, we observed that a signature comprising the candidate genes identified in the screen was up-regulated in senescence (Fig. 2C; Supplemental Fig. S2b).

To validate the screen results, we devised a secondary screen that took advantage of IMR90 cells expressing TRF2^ΔBΔM^ in a doxycycline-inducible fashion (Fig. 2D; Supplemental Fig. S2c,d). Induction of TRF2^ΔBΔM^ resulted in a swift growth arrest that was evident by a drop in BrdU incorporation and could be prevented by transfection with siRNAs targeting p53 (Supplemental Fig. S2e). We designed four independent siRNAs against each of the candidate genes and screened them using this system. Candidates were then ranked based on B-score (Fig. 2E). The top candidate identified in this secondary screen was Exportin 7 (Supplemental Fig. S2f), a protein involved in both nuclear import and export (Aksu et al. 2018). Using two independent siRNAs, we confirmed that knockdown of XPO7 resulted in increased BrdU incorporation and diminished induction of p21^CIP1^ during senescence (Fig. 2F).

### Knockdown of XPO7 blunts senescence

**Figure 3.**
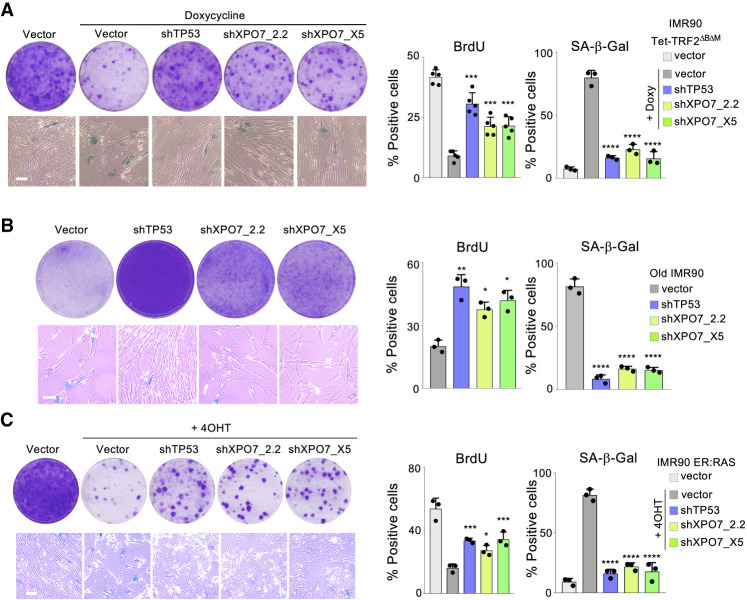
XPO7 depletion prevents senescence. (*A*) TRF2^ΔBΔM^-induced senescence. Representative images of colony formation assay (crystal violet, *top left*) and SA-β-galactosidase (*bottom left*) in IMR90 Tet-TRF2^ΔBΔM^ cells infected with the indicated vectors. Quantification of the percentage of BrdU-positive (*middle*, *n* = 5 independent experiments) and SA-β-galactosidase-positive (*right*, *n* = 3 independent experiments) cells. (*B*) Replicative senescence. Representative images of colony formation assay (crystal violet, *top left*) and SA-β-galactosidase (*bottom left*) in old IMR90 cells (passage 24) infected with the indicated vectors. Quantification of the percentage of BrdU-positive (*middle*) and SA-β-galactosidase-positive (right) cells (*n* = 3 independent experiments). (*C*) Oncogene-induced senescence. Representative images of colony formation assay (crystal violet, *top left*) and SA-β-galactosidase (*bottom left*) in IMR90 ER:RAS cells infected with the indicated vectors. Quantification of the percentage of BrdU-positive (*middle*) and SA-β-galactosidase-positive (*right*) cells (*n* = 3 independent experiments). All scale bars, 50 µm. (****) *P* < 0.0001, (***) *P* < 0.001, (**) *P* < 0.01, (*) *P* < 0.05. All statistical significances were calculated using one-way ANOVA with multiple comparison with control senescent cells for each condition.

To further study the role of XPO7 in senescence, we cloned three shRNAs that knocked down XPO7 expression (Supplemental Fig. S3a,b) and analyzed the effect that XPO7 depletion had in senescence. Similar to what we observed using siRNAs, we validated that XPO7 knockdown partially prevented senescence caused by TRF2^ΔBΔM^ induction, as noted by increased growth and BrdU incorporation and decreased SA-β-Gal activity (Fig. 3A). To understand whether the effect of XPO7 was limited to regulating TRF2^ΔBΔM^-induced senescence or XPO7 was a wider regulator of senescence, we analyzed the effect on replicative senescence. We knocked down XPO7 expression in late-passage IMR90 cells, observing that XPO7 knockdown blunted replicative senescence (Fig. 3B). Interestingly, depletion of XPO7 in early-passage IMR90 cells resulted in increased proliferation (Supplemental Fig. S3c). Moreover, depletion of XPO7 also modulated irradiation-induced senescence (Supplemental Fig. S3d) and oncogene-induced senescence, as we observed by using IMR90 ER:RAS cells (Fig. 3C), a widely employed model of OIS (Acosta et al. 2013). In summary the above results confirm that XPO7 is a general regulator of senescence.

### XPO7 regulates p21^CIP1^ to control oncogene-induced senescence

To investigate how *XPO7* could regulate senescence, we first analyzed publicly available data on gene dependency derived from CRISPR screens (Meyers et al. 2017). Using the DepMap portal (https://depmap.org), we searched for genes whose knockout best correlated with that of XPO7. Interestingly, nine out of the top 10 genes showing the best correlation belonged to the p53 pathway (Supplemental Fig. S4a). These included *TP53* (encoding for p53) (Supplemental Fig. S4b) and *CDKN1A* (encoding for p21^CIP1^) (Supplemental Fig. S4c). In contrast, the correlation between *XPO7* and CDKN2A (encoding for p16^INK4a^) (Supplemental Fig. S4d) was weaker. Moreover, the correlation between *XPO7* and *TP53* was driven by TP53 wt cells (Supplemental Fig. S4e). These data suggest that XPO7 might regulate some component of the p53 pathway.

**Figure 4.**
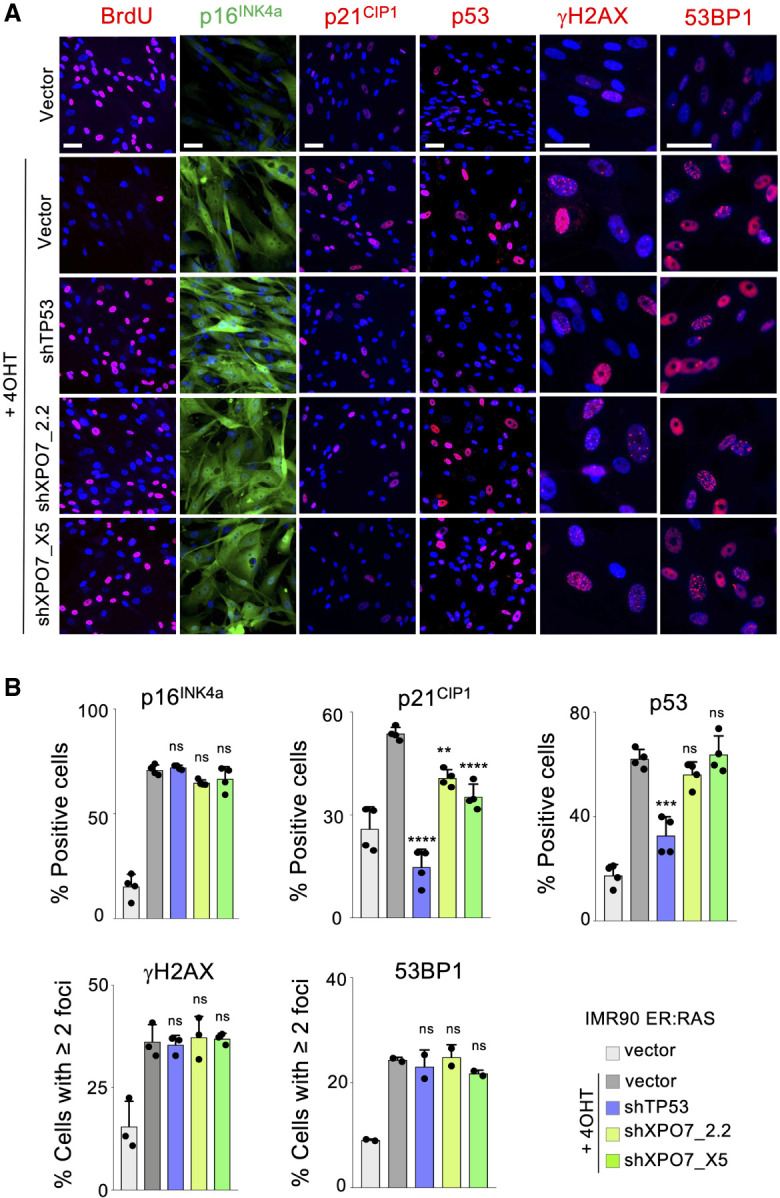
XPO7 regulates p21^CIP1^ to control oncogene-induced senescence. IMR90 ER:Ras cells transduced with shRNA targeting *TP53*, *XPO7* or a nontargeting control (vector) and treated with 4OHT were indicated to induce OIS. (*A*) Representative images of immunofluorescence against BrdU, p16^INK4a^, p21^CIP1^, p53, γH2AX, and 53BP1. Scale bars, 50 µm. (*B*) Quantification of p16^INK4a^ (*n* = 4 independent experiments), p21^CIP1^ (*n* = 4 independent experiments), p53 (*n* = 4 independent experiments), γH2AX (*n* = 3 independent experiments), and 53BP1 (*n* = 2 independent experiments). (****) *P* < 0.0001, (***) *P* < 0.001, (**) *P* < 0.01, (n.s.) nonsignificant. All statistical significances were calculated using one-way ANOVA with multiple comparisons with control senescent cells for each condition. All error bars represent mean ± SD.

To better understand how XPO7 controls senescence, we next analyzed how XPO7 knockdown affected the expression of different effectors of OIS, using immunofluorescence assays. XPO7 knockdown did not result in reduced DNA damage or changes in the levels of p53 during OIS (Fig. 4). Interestingly, we observed that knocking down XPO7 partially prevented the induction of p21^CIP1^, but it did not affect the expression of other cyclin-dependent kinase inhibitors such as p16^INK4a^ during OIS (Fig. 4A,B). This regulation was also observed using immunoblots and at the mRNA level (Supplemental Fig. S3a,b. The above results suggest that XPO7 regulates senescence by affecting p21^CIP1^ induction.

### XPO7 regulates the nuclear levels of TCF3 to control senescence

**Figure 5.**
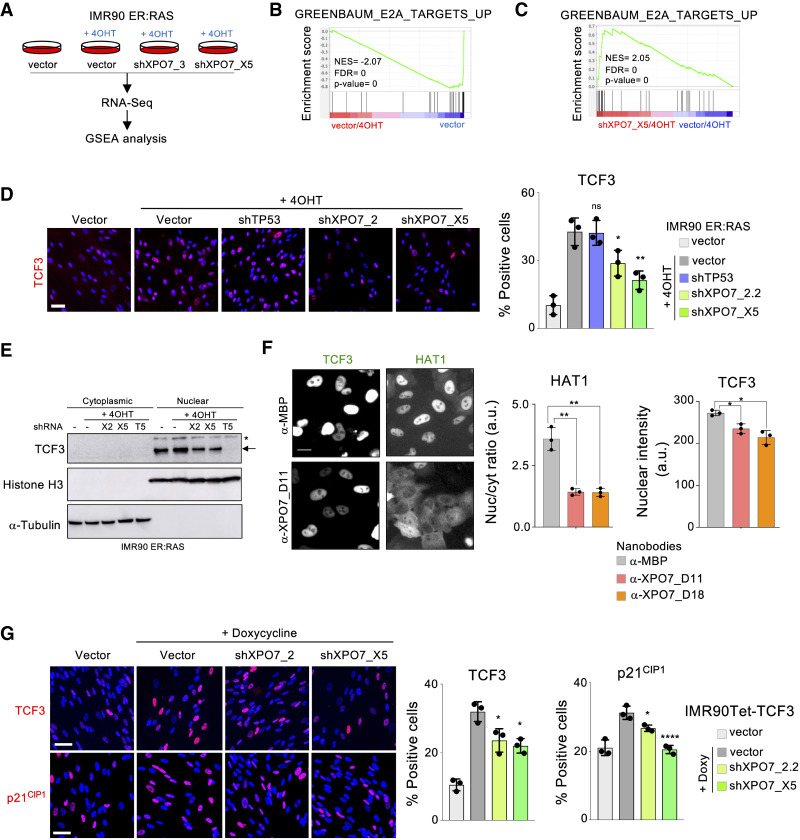
XPO7 regulates nuclear levels of TCF3 to control senescence. (*A*) RNA sequencing (RNA-seq) was performed with IMR90 ER:RAS cells transduced with the indicated vectors. When noted, 4OHT was added to induce OIS. Data were subjected to gene set enrichment analysis (GSEA). (*B*,*C*) GSEA showing depletion of a signature associated with the knockdown of TCF3 target genes (Greenbaum_E2A_targets_up) during OIS (vector/4OHT vs. vector) (*B*) and its enrichment in cells undergoing senescence infected with XPO7 shRNAs (shXPO7_X5/4OHT vs. vector/4OHT) (*C*). (NES) Normalized enriched score, (FDR) false discovery rate. (*D*) XPO7 prevents nuclear TCF3 accumulation during OIS. IMR90 ER:RAS cells were transduced with the indicated vectors and treated with 4OHT where indicated to induce OIS. Representative pictures of TCF3 IF (*left*) and quantification (*right*) are shown (*n* = 3 independent experiments). (**) *P* < 0.01, (*) *P* < 0.05, (n.s.) nonsignificant. All statistical significances were calculated using one-way ANOVA with multiple comparisons with control senescent cells for each condition. All error bars represent mean ± SD. (*E*) Immunoblot to analyze the distribution of TCF3 in cytosolic and nuclear fractions. IMR90 ER:RAS cells were transduced with shRNAs targeting XPO7 ([X2] XPO7_2.2; [X5] XPO7_X5), TCF3 ([T5] TCF3_G5) or a nontargeting control (−). Senescence was induced in IMR90 ER:RAS cells by adding 4OHT where indicated. An arrow indicates the correct band for TCF3, while the asterisk indicates a nonspecific band. Histone H3 and α-tubulin were used as markers for the nuclear and cytosolic fractions, respectively. (*F*) GFP-fused TCF3 or HAT1 were transfected in HeLa cells together with nanobodies recognizing MBP or XPO7. IF was conducted to assess their subcellular localization. (*Left*) Representative pictures are shown. Scale bar, 20 μm. Nuclear/cytoplasmic GFP ratio for GFP-HAT1 (*middle*) and nuclear intensity for TCF3-GFP (*right*) are shown (*n* = 3 independent experiments). (**) *P* < 0.01, (*) *P* < 0.05. All statistical significances were calculated using an unpaired two-sided *t*-test. (*G*) Inducible expression of TCF3. IMR90 Tet-TCF3 cells were transduced with shRNA targeting XPO7 or a nontargeting control (vector) and treated with 100 ng/mL doxycycline to induce TCF3 expression. (*Left*) Representative pictures of TCF3 (*top*) and p21^CIP1^ (*bottom*) immunofluorescence. Scale bars, 50 µm. (*Right*) Quantification from *n* = 3 independent experiments. (****) *P* < 0.0001, (*) *P* < 0.05. Statistical significance was calculated using one-way ANOVA with multiple comparisons with control senescent cells. All error bars represent mean ± SD.

To further investigate how XPO7 regulates senescence, we performed transcriptional profiling of IMR90 ER:RAS cells infected with two independent shRNAs targeting XPO7 (Fig. 5A; Supplemental Fig. S5a). Taking advantage of GSEA, we observed that signatures of OIS were significantly down-regulated upon XPO7 knockdown (Supplemental Fig. S5b–d). We also observed an association between the transcriptome of XPO7-depleted cells and TCF3-dependent signatures (Fig. 5B,C; Supplemental Fig. S5e). Interestingly it has been previously suggested that the transcription factor E2A/TCF3 is shuttled by XPO7 (Lee et al. 2010). TCF3 has also been shown to regulate p21^CIP1^ levels. (Andrysik et al. 2013).

We took advantage of quantitative IF to understand how the levels of TCF3 changed during OIS. Nuclear TCF3 levels increased during OIS, and this increase was prevented upon XPO7 knockdown (Fig. 5D; Supplemental Fig. S6a). While subcellular fractionation confirmed reduced levels of TCF3 in the nuclei, we could not detect TCF3 in the cytoplasmic fraction (Fig. 5E). To understand whether XPO7 regulated the subcellular localization of TCF3 or rather affected its levels by other mechanisms, we used a system (Supplemental Fig. S6b,c) that combines GFP fusion proteins with intracellular XPO7 nanobodies. The nanobodies can sequester XPO7 and affect the subcellular localization of its shuttling substrates such as HAT1 (Aksu et al. 2018). Indeed, XPO7 nanobodies blocked the nuclear import of HAT1. However, nanobodies targeting XPO7 caused reduced TCF3 nuclear levels but without any increase in its cytoplasmic localization (Fig. 5F; Supplemental Fig. S6d). Knockdown of XPO7 also reduced the nuclear levels of ectopically expressed TCF3, suggesting that XPO7 affects TCF3 levels via a post-transcriptional mechanism. Interestingly, XPO7 knockdown also blunted the up-regulation of p21^CIP1^ caused by ectopic expression of TCF3 (Fig. 5G). Overall, these results suggest that XPO7 does not regulate TCF3 localization but rather its overall levels.

### TCF3 regulates the expression of p21^CIP1^ during OIS

**Figure 6.**
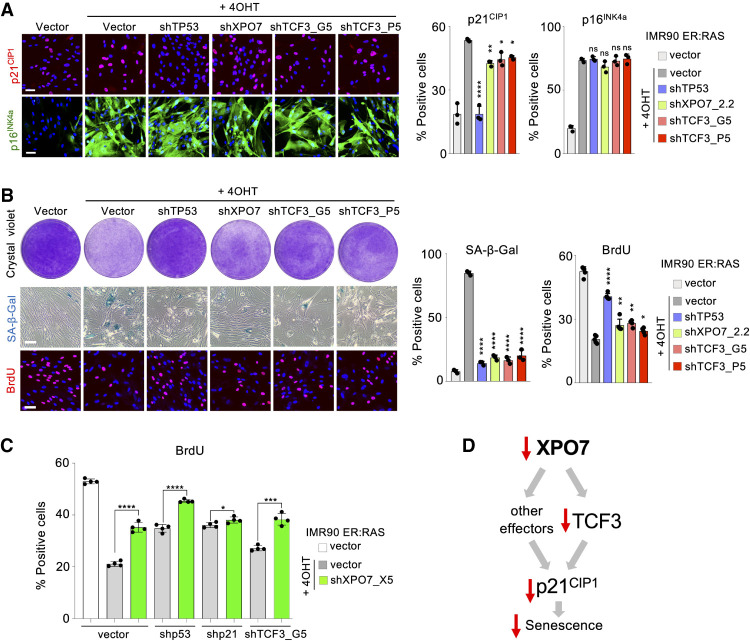
TCF3 controls p21^CIP1^ levels during OIS. IMR90 ER:Ras cells transduced with shRNA targeting *TP53*, *XPO7*, *TCF3*, or a nontargeting control (vector) and treated with 4OHT when indicated to induce OIS. (*A*) Representative images of p21^CIP1^ and p16^INK4a^ IF (*left*) and quantification of the percentage of cells positive for p21^CIP1^ (*middle*) and p16^INK4a^ (*right*). (*B*) Representative images for colony formation assays (crystal violet, *top left*), SA-β-galactosidase staining (*middle left*), and BrdU staining (*bottom left*). Quantification of the percentage of cells positive for SA-β-galactosidase staining (*middle*) and BrdU (*right*). Scale bars, 50 µm (*n* = 3 independent experiments). (****) *P* < 0.0001, (**) *P* < 0.01, (*) *P* < 0.05, (n.s.) nonsignificant. Statistical significance was calculated using one-way ANOVA with multiple comparisons with control senescent cells. All error bars represent mean ± SD. (*C*) Effect of XPO7 knockdown in shp53, shp21, and shTCF3 cells. IMR90 ER:Ras cells transduced with shRNA targeting *TP53*, *CDKN1A* (encoding for p21), *TCF3*, or a nontargeting control (vector) were infected with a shRNA targeting *XPO7* or a nontargeting control (vector) and treated with 4OHT were indicated to induce OIS. Quantification of the percentage of cells positive for BrdU is shown (*n* = 4 independent experiments). (****) *P* < 0.0001, (***) *P* < 0.001, (*) *P* < 0.05. Statistical significance was calculated using an unpaired, two-tailed Student's *t*-test. All error bars represent mean ± SD. (*D*) Scheme summarizing how XPO7 regulates senescence.

To understand whether TCF3 regulates p21^CIP1^ during OIS, we generated two shRNAs that knocked down TCF3 expression (Supplemental Fig. S7a–c). Using these shRNAs, we observed that depletion of TCF3 partially prevented the up-regulation of p21^CIP1^ without affecting p16^INK4a^ induction during OIS (Fig. 6A). Moreover, TCF3 knockdown partially rescued senescence induction as assessed by SA-β-Gal staining, BrdU incorporation, and colony formation assays (Fig. 6B). To better understand how XPO7 regulates senescence, we tested how XPO7 knockdown affected cells in which p53, p21^CIP1^, or TCF3 have been depleted. While XPO7 depletion resulted in a substantial increase of BrdU incorporation in both p53- and TCF3-depleted cells, that was not the case in p21-depleted cells (Fig. 6C; Supplemental Fig. S7d). Overall, our results show that p21^CIP1^ is key to explain how XPO7 affects senescence and suggest that TCF3 is not the only target controlled by XPO7 regulating p21^CIP1^-mediated senescence (Fig. 6D).

### *XPO7* is a tumor suppressor gene that regulates OIS

**Figure 7.**
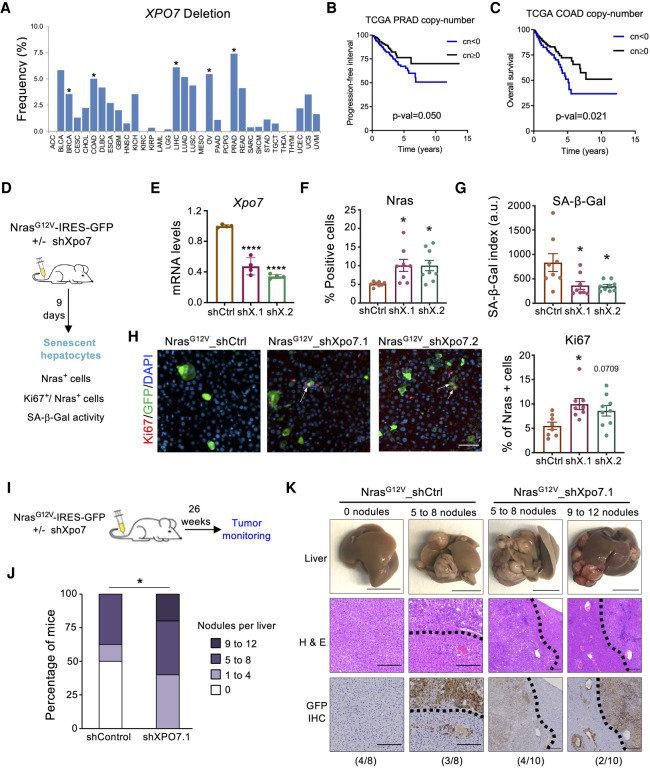
*XPO7* is a tumor suppressor gene that regulates OIS. (*A*) Frequency of XPO7 deletions in 33 cancer types from the Pan-Cancer Atlas of the TCGA consortium. Asterisks indicate cohorts where deep deletions are significant (https://portals.broadinstitute.org/tcga/home). Samples are ordered alphabetically from *left* to *right*. (*B*,*C*) Survival curves from the prostate adenocarcinoma (*B*) and colon (*C*) cohorts (PRAD and COAD, respectively) within TCGA. Patients were stratified depending on XPO7 copy number values on their tumors. Tumors having absolute numbers −2 (deep deletion) or −1 (shallow deletion) were grouped together and represented versus remaining tumors. *P*-values are calculated using a log rank test. Patients having low XPO7 copy number display a worse prognosis. (*D*) Schematic of hydrodynamic tail vein injection (HTVI) experiments shown in *E*–*H*. A transposon plasmid encoding Nras^G12V^ and Xpo7 shRNA was coinjected with a plasmid expressing transposase (*n* = 8 mice for shCtrl, *n* = 8 for mice shXpo7.1, and *n* = 9 mice for shXpo7.2). (*E*) The expression of *Xpo7*, was determined using RT–qPCR (*n* = 4 independent experiments). (*F*) Quantification of the GFP-positive cells (as a surrogate of NRas^G12V^-positive cells). (*G*) Quantifications of SA-β-galactosidase activity. Scale bar, 50 μm. (*H*, *left*) Immunofluorescence staining of GFP (green) and Ki67 (red) in liver sections. (*Right*) Quantifications of the percentage of GFP-positive cells (as a surrogate of NRas^G12V^-positive cells) that are also positive for Ki67. Scale bar, 20 μm. (*) *P* < 0.05. All statistical significances were calculated using one-way ANOVA (Dunnett's test) compared with shCtrl. Error bars represent mean ± SD for *E* and mean ± SEM for *F*–*H*. (*I*) Schematic of HTVI tumor monitoring experiments shown in *J*,*K*. Mice injected with plasmids were maintained for 26 wk to monitor tumorigenesis (*n* = 8 mice for shCtrl and *n* = 10 mice for shXpo7.1). (*J*) Quantifications of nodule numbers in livers. Percentage of mice with different nodule numbers are shown. Statistical significance was calculated using Fisher's exact test of the tumor versus nontumor mice in the shControl versus shXPO7.1 cohorts. (*K*) Livers were analyzed for nodular tumors (*top*), H&E staining (*middle*), and immunohistochemistry staining of GFP (*bottom*). Scale bars: 1 cm for liver, 100 μm for H&E and GFP IHC. (*) *P* < 0.05. Error bars represent mean ± SD for *E* and mean ± SEM for *F*–*H*.

Senescence escape is a hallmark of cancer (Hanahan and Weinberg 2011). Therefore, genes that regulate senescence are often altered in tumors. To understand whether *XPO7* has the characteristics of a tumor suppressor gene, we investigated the status of *XPO7* in tumor samples in the Cancer Genome Atlas (TCGA) program (https://www.cancer.gov/tcga). While we did not observe a significant number of tumors presenting amplifications of the *XPO7* gene, deletions and mutations were more common (Fig. 7A; Supplemental Fig. S8a,b). In particular, deletions of *XPO7* occurred in a significant percentage of samples obtained from different tumor types, including prostate adenocarcinomas (PRAD), colon adenocarcinomas (COAD), or liver hepatocellular carcinomas (LIHC), among others (Fig. 7A). Reinforcing the hypothesis that XPO7 could be a novel tumor suppressor, PRAD patients with heterozygous or homozygous deletion of *XPO7* had a significantly worse progression-free survival than their wt counterparts (Fig. 7B). Similarly, *XPO7* loss correlated with worse overall survival in COAD patients (Fig. 7C). Moreover, not only deletion of the *XPO7* gene but also reduced XPO7 expression correlated with worse prognosis in a range of tumors that included PRAD, COAD, (Supplemental Fig. S8c,d) and acute myeloid leukemias (AML) (Supplemental Fig. S8e; Metzeler et al. 2008). We also observed a correlation between XPO7 expression and copy number in PRAD and COAD (Supplemental Fig. S8f,g). Consistent with the key role for p21^CIP1^ as a mediator of XPO7, COAD tumors with homozygous *XPO7* deletion expressed lower levels of *CDKN1A* than *XPO7* wt tumors (Supplemental Fig. S8h).

Next, we asked whether XPO7 depletion could prevent senescence in vivo, as that could explain its role in tumorigenesis. Since hepatocellular carcinomas are one of the tumor types in which we observed significant deletions of *XPO7* (Fig. 7A), we took advantage of a liver model of OIS (Kang et al. 2011). In this model, OIS is triggered in hepatocytes via transposon-mediated transfer of oncogenic Nras (Nras^G12V^) delivered by hydrodynamic tail vein injection (HTVI) (Fig. 7D). The senescent hepatocytes activate an immune response that results in their gradual elimination (Kang et al. 2011). To investigate whether Xpo7 knockdown affects OIS in vivo, we generated vectors that coexpressed Nras^G12V^ and either control (ctrl) or *Xpo7* targeting shRNAs (Fig. 7E). At day 9 postinjection, there were more Nras^G12V^-positive hepatocytes present in the livers of mice transduced with the shRNAs targeting *Xpo7* compared with shControls (Fig. 7F; Supplemental Fig. S9a). The higher percentage of Nras+ hepatocytes correlated with reduced senescence, as shown by decreased levels of SA-β-Gal in the livers of mice transduced with Nras^G12V^_shXpo7 vectors (Fig. 7G; Supplemental Fig. S9b). In addition, knockdown of *Xpo7* resulted in a higher percentage of proliferating Nras-positive hepatocytes, as indicated by costaining with Ki67 (Fig. 7H). Therefore, depletion of *Xpo7* blunted senescence in vivo and resulted in a higher percentage of proliferating Nras+ hepatocytes. We then investigated whether knockdown of *Xpo7* cooperated with Nras^G12V^ to induce malignant transformation. To test this, we used a similar set-up and monitored mice for tumor formation after 26 wk (Fig. 7I). While four out of eight control mice displayed liver nodular tumors, all (10 out of 10) shXpo7.1 mice had hepatocellular carcinoma (Fig. 7J; Supplemental Fig. S9a,b). Immunohistochemistry confirmed that the tumors derived from hepatocytes transduced with the vectors, as they were GFP-positive (Fig. 7K). While there was heterogeneity, a subset of mice injected with Nras^G12V^_shXpo7.1 displayed more liver nodules than their Nras^G12V^_shControl counterparts (Fig. 7J,K; Supplemental Fig. S9a,b). Altogether, these results indicate that XPO7 is a novel tumor suppressor gene that functions by regulating senescence.

## Discussion

Given the intricate relationship between senescence and cancer, we reasoned that genes whose knockdown prevented senescence could be potential tumor suppressors. Since telomeric DNA damage is common to many types of senescence, including replicative and oncogene-induced senescence, we took advantage of a previously described model for senescence caused by telomere dysfunction (Jacobs and de Lange 2004). Expression of a dominant negative version of the shelterin component TRF2 (TRF2^ΔBΔM^) is sufficient to induce senescence (Jacobs and de Lange 2004). Our screen identified several components of the DNA damage or DNA repair pathways. More interestingly, it also unveiled a cluster of proteins involved in intracellular transport as regulators of senescence. Among those, we identified two nuclear cytoplasmic transporters, RANBP17 and RANBP16/XPO7 (Kutay et al. 2000; Mingot et al. 2004).

Our secondary screen and subsequent experiments confirmed that depletion of XPO7 blunts senescence induction. XPO7 acts not only as an exportin but also as a nuclear import receptor (Aksu et al. 2018). Little is known about XPO7 function. Its best characterized role is in the regulation of erythropoiesis (Hattangadi et al. 2014); however, it is unclear whether this role relates to XPO7-mediated nuclear export. To understand how XPO7 could regulate senescence, we first looked into DepMap (http://depmap.org), a public repository for CRISPR screens. Interestingly we found a positive correlation between the effects of knocking out *XPO7* and the effects of knocking out *TP53*, *CDKN1A*, and other effectors or positive regulators of the p53 pathway. These data suggested the existence of an epistatic relation between XPO7 and the p53 pathway. Further analysis showed that XPO7 depletion regulates senescence by blunting the transcriptional up-regulation of p21^CIP1^. Given that XPO7 controls shuttling of proteins between the nuclei and the cytoplasm, we investigated its known substrates. A recent work identified ∼200 export cargoes and ∼30 nuclear import substrates of XPO7 (Aksu et al. 2018). In addition, XPO7 also regulates NF-κB translocation to the nucleus (Liang et al. 2013), while both XPO7 and RANBP17 can interact with E2A/TCF3, regulating their function, although it is uncertain whether this role relates to nuclear shuttling (Lee et al. 2010). Indeed, GSEA showed us that TCF3 activity increases during oncogene-induced senescence in a manner dependent upon XPO7. We decided to investigate this relation further since it has also been described that TCF3 controls p21^CIP1^ transcription (Andrysik et al. 2013). We observed that nuclear levels of TCF3 increased during OIS in an XPO7-dependent way and that TCF3 contributes to regulate p21^CIP1^ expression during senescence. However, our results suggest that, rather than regulating TCF3 localization, XPO7 seems to affect its levels by a mechanism not yet understood. In addition, factors other than TCF3 might contribute to explain the role of XPO7 in regulating senescence. Overall, our results support an important role for both TCF3 and p21^CIP1^ in the process (Fig. 6D).

Given the ability of XPO7 to control senescence, we searched for evidence of a role of XPO7 in cancer. Data derived from the TCGA unveiled different cancer types (including tumors of the liver, colon, or prostate) in which deletions of XPO7 are significant. Moreover, low expression of XPO7 or *XPO7* loss correlated with worse outcome in cancers of the prostate or the colon. Low XPO7 expression also correlated with a worse outcome in AML. This is interesting, since a CRISPR screen looking for genes essential for AML identified that sgRNAs targeting XPO7 confer a growth advantage (Yamauchi et al. 2018), which is consistent with the results reported here. We provided further evidence for the dual role of XPO7 in regulating senescence and tumor suppression using a mouse model of OIS and tumor initiation in liver cancer (Kang et al. 2011). In this context, XPO7 depletion results in reduced p21^CIP1^ levels, blunted OIS, and cooperated with oncogenic Nras^G12V^ to cause liver tumors.

In summary, we identified the nuclear exportin XPO7 as a novel regulator of senescence and tumor suppression. Our work provides a basis to elucidate the role of XPO7 in cancer. Moreover, it will be interesting to investigate whether tumors with *XPO7* deletion or expressing low levels of XPO7 are subject to dependencies that could be exploited therapeutically.

## Materials and methods

### Plasmids

Plasmids used in this study are listed in Supplemental Table S1. Plasmids used for the nanobody experiments have been described in Aksu et al. (2018). For pEGFP-TCF3, we amplified TCF3 cDNA from pcDNA3 hE47 (Addgene) (Jen et al. 1992), using the following primers: CCGGAATTCACCGCCATGAACCAGCCGCAGAG and GCCGGGCACATGTGAGGATCCGCC. The corresponding product was digested with EcoRI and BamHI and cloned into pEGFP-C1. The sequences targeted by shRNA vectors are listed in Supplemental Table S2.

### Transfection of siRNAs

IMR90-tet-TRF2^ΔBΔM^ cells in suspension (100 µL) were reverse-transfected with siRNAs (Qiagen) in a well of a 96-well plate. The suspension media was DMEM supplemented with 10% FBS (tetracycline free) only. The transfection mix for each sample contained 0.1 µL of DharmaFECT^TM^ 1 (Dharmacon) in 17.4 µL plain DMEM mixed with 3.6 µL siRNA (final concentration 30 nM) 30 min before cell seeding. Eighteen hours after transfection, the media was replaced with fresh complete media containing 100 ng/mL doxycycline to induce TRF2^ΔBΔM^ expression. The cells were fixed 72 h after transfection with 4% PFA (w/v) before immunofluorescence staining. In the case of BrdU measurement, the culture medium was supplemented with 10 µM BrdU for 16–18 h prior to fixation. The siRNAs used in this study are listed in Supplemental Table S3.

### Primary shRNA and secondary siRNA screens

Details of primary shRNA and secondary siRNA screens are in the Supplemental Material.

### Growth assays

BrdU incorporation and colony formation assays with crystal violet were performed as previously described (Georgilis et al. 2018). Briefly, for BrdU incorporation assays, the cells were incubated with 10 µM BrdU for 16–18 h before being fixed with 4% (w/v) PFA. BrdU incorporation was assessed by immunofluorescence and high content analysis microscopy. For crystal violet staining, the cells were seeded at low density on six-well dishes or 10-cm plates and fixed at the end of the treatment with 0.5% (w/v) glutaraldehyde. The plates were then stained with 0.2% crystal violet.

### Cytochemical SA-β-galactosidase assay

Cells were grown in six-well plates until day 7, fixed with 0.5% (w/v) glutaraldehyde (Sigma) in PBS for 10–15 min, washed with 1 mM MgCl_2_/PBS (pH 6.0), and then incubated with X-Gal staining solution (1 mg/mL X-Gal, Thermo Scientific), 5 mM K_3_[Fe(CN)_6_], and 5 mM K_4_[Fe(CN)_6_] for 8 h at 37°C. Bright-field images of cells were taken using a DP20 digital camera attached to the Olympus CKX41 inverted light microscope. The percentage of SA-β-Gal-positive cells was estimated by counting at least 100 cells per replicate.

### Immunofluorescence staining of cells for high content analysis

Cells were grown in 96-well plates, fixed with 4% (w/v) PFA, and stained as previously described (Georgilis et al. 2018). Antibodies are listed in Supplemental Table S4.

### XPO7 alterations in the Pan-Cancer Atlas of the TCGA consortium

Frequencies for *XPO7* copy number alterations (amplification or deletion) and mutations were retrieved from the cBioportal webpage (https://www.cbioportal.org) for each of the 33 cancer types analyzed. Significant copy number alterations were calculated using the “Browse GISTIC Data By Gene” utility within the TCGA copy number portal of the Broad Institute (https://portals.broadinstitute.org/tcga/home).

### XPO7 alterations and survival analysis

Clinical outcomes from TCGA patients were retrieved from Liu et al. (2018). Survival analysis was performed in GraphPad Prism 7. Briefly, patients were stratified depending on *XPO7* copy number values or mRNA expression in their tumors. For copy number, tumors having absolute numbers −2 (deep deletion) or −1 (shallow deletion) were grouped together and represented versus remaining tumors. For mRNA expression, tumors were ranked from lowest to highest mRNA values, and three groups were analyzed corresponding to three tertiles, from lowest (Tertile 1) to highest (Tertile 3) values. *P*-values were calculated using a log rank test.

### Gene expression analysis

Total RNA was extracted using Trizol reagent (Invitrogen) and the RNeasy isolation kit (Qiagen). cDNA was generated using random hexamers and SuperScript II reverse transcriptase (Invitrogen). Quantitative real-time PCR was performed using SYBR Green PCR master mix (Applied Biosystems) in a CFX96 real-time PCR detection system (Bio-Rad). *GAPDH* or *RPS14* expression were used for normalization. Primers are listed in Supplemental Table S5.

### In vivo induction of OIS in hepatocytes

Five-week-old female C57BL/6 mice (Charles River) were used for hydrodynamic tail vein injection (Kang et al. 2011). All plasmids were prepared with GenElute HP Endotoxin-Free Maxiprep kit (Sigma). Twenty-five micrograms of shRNA-expressing CaNIGmiRE vector and 5 µg of transposase-encoding vector were diluted in normal saline to a final volume of 10% body weight. HDTVI was performed within 7–8 sec, and the livers were collected at the indicated time. All animal procedures were carried out with Imperial College London Research Ethics Committee approval in accordance with the Animals (Scientific Procedures) Act of 1986 (UK).

### Immunoblot

Cells were lysed in RIPA buffer (80 mM Tris at pH 8.0, 150 mM NaCl, 1% Triton X-100, 0.5% Na-Doc, 0.1% SDS, 1 mM EDTA) supplemented with one tablet of phosphatase and one tablet of protease inhibitors (Roche) per 10 mL of RIPA. Immunoblotting was carried out as previously described (Georgilis et al. 2018). Antibodies are listed in Supplemental Table S4.

### Subcellular fractionation

For subcellular fractionation, a nuclear extract kit (Active Motif) was used. In brief, cells were trypsinized, washed with ice-cold PBS, and resuspended in hypotonic buffer. After 30 min., detergent was added, and cells were vortexed briefly to release nuclei. After centrifugation, supernatant (cytosolic fraction) was collected, and nuclei were washed with hypotonic buffer. Nuclei were lysed with RIPA buffer. For protein quantification, a Pierce BCA protein assay kit (Thermo Scientific) was used.

### Experiments with XPO7 nanobodies

These experiments were carried out as described in Aksu et al. (2018). Briefly, HeLa cells were cotransfected with plasmids expressing the GFP fusion protein and nanobodies in 96-well plates using 0.75 µL of PEI and 50 ng of each plasmid per well. Cells were fixed 48 h after transfection and GFP fluorescence imaged using an InCell 2000 high-throughput automated microscope. Hoechst (Sigma) was used for nuclear counterstaining.

### Statistical analysis

GraphPad Prism 8.0 was used for statistical analysis. The statistical tests used are referred to in the figure legends. Values are presented as mean ± SD unless otherwise indicated. Asterisks always indicate significant differences as follows: not significant (ns), *P* < 0.5 (*), *P* < 0.01 (**), and *P* < 0.001 (***). For in vivo studies, mice were randomly assigned to treatment groups. All replicates in this study represent different mice.

### Accession numbers

The RNA-seq (GSE153921) and microarray (GSE153922) data sets described in this work have been deposited at the Gene Expression Omnibus.

### Competing interest statement

J.G. has acted as a consultant for Unity Biotechnology, Geras Bio, Myricx Pharma, and Merck KGaA; owns equity in Unity Biotechnology and Geras Bio; and is a named inventor in Imperial College and MRC patents related to senolytic therapies (not related to the work presented here).

## Supplementary Material

Supplemental Material
