# Supplementary material for: XPO7 is a tumor suppressor regulating p21CIP1-dependent senescence
Source: Genes Dev. 2021 Mar 1;35(5-6):379–91. doi: 10.1101/gad.343269.120 (PMC7919420; doi:10.1101/gad.343269.120)
Supplement: Supplemental Material [file supp_35_5-6_379__index.html]

XPO7 is a tumor suppressor regulating p21CIP1-dependent senescence — Supplemental Material 

# XPO7 is a tumor suppressor regulating p21CIP1-dependent senescence

## Supplemental Material

- Supplemental\_Data\_Innes343269.pdf
