# Supplementary material for: XPO7 is a tumor suppressor regulating p21CIP1-dependent senescence
Source: Genes Dev. 2021 Mar 1;35(5-6):379–91. doi: 10.1101/gad.343269.120 (PMC7919420; doi:10.1101/gad.343269.120)
Supplement: Supplemental Material [file supp_gad.343269.120_Supplemental_Data_Innes343269.pdf]

SUPPLEMENTAL DATA for

XPO7 is a tumour suppressor regulating p21^{CIP1}-dependent senescence

Andrew J. Innes, Bin Sun, Verena Wagner, Sharon Brookes, Domhnall McHugh, Joaquim Pombo, Rosa María Porreca, Gopuraja Dharmalingam, Santiago Vernia, Johannes Zuber, Jean-Baptiste Vannier, Ramón García-Escudero and Jesús Gil

Including:

- **Supplemental Figures S1 to S9 and legends**
- **Supplemental Tables S1 to S5**
- **Supplemental Materials and Methods**
- **Supplemental References**

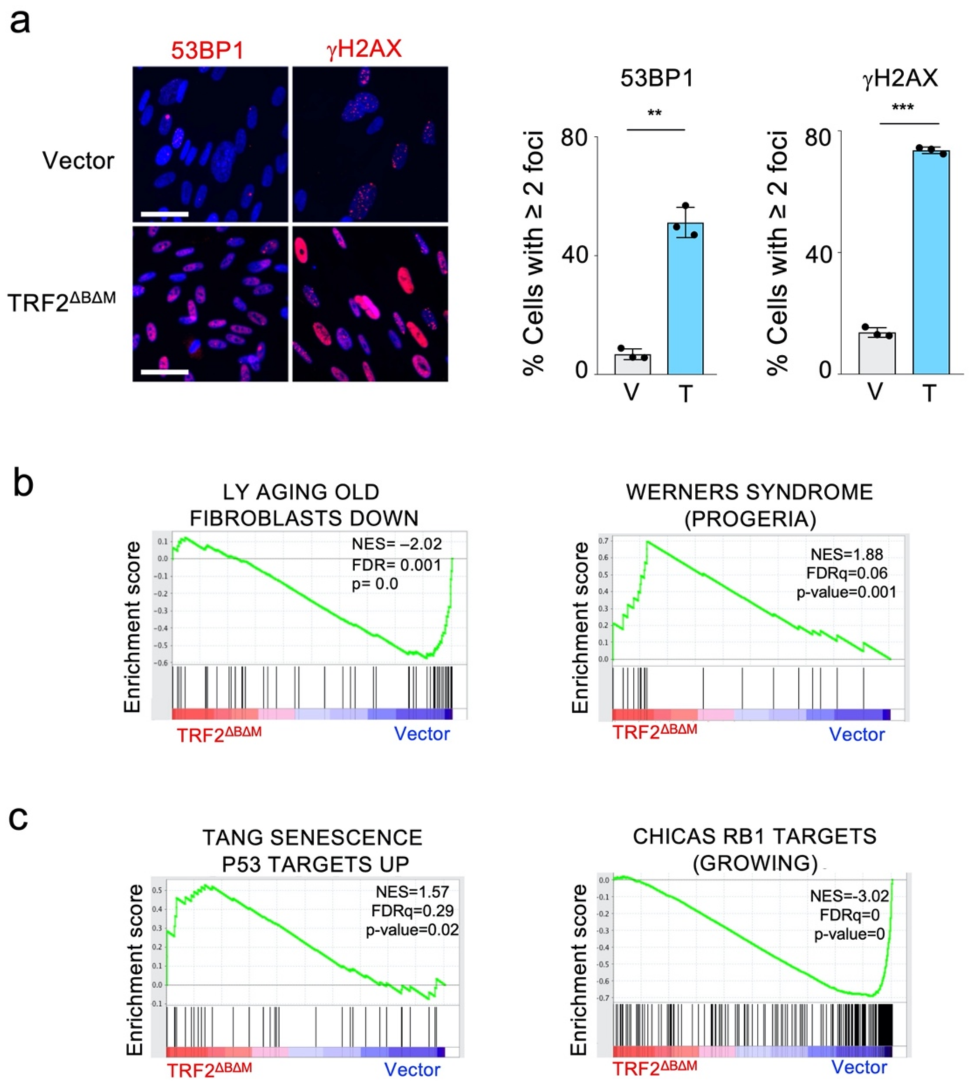

Supplemental Figure S1. Expression of TRF2$^{\Delta B\Delta M}$ induces senescence in IMR90 cells.

(a) IMR90 cells were infected with an empty vector (V) or a vector expressing TRF2$^{\Delta B\Delta M}$ (T). Representative images of IF against 53BP1 or γH2AX (left). Scale bars represent 20 µm. Percentage of cells with 2 or more 53BP1 (middle) or γH2AX (right) foci are shown. (*n* = 3 independent experiments). ***P<0.001, **P<0.01. Unpaired, two-tailed, Student's t-test. All error bars represent mean ± s.d. (b-c) GSEA showing the enrichment of different signature in the gene expression profile of IMR90 TRF2$^{\Delta B\Delta M}$ versus IMR90 vector cells. NES, normalized enriched score; FDR, false discovery rate.

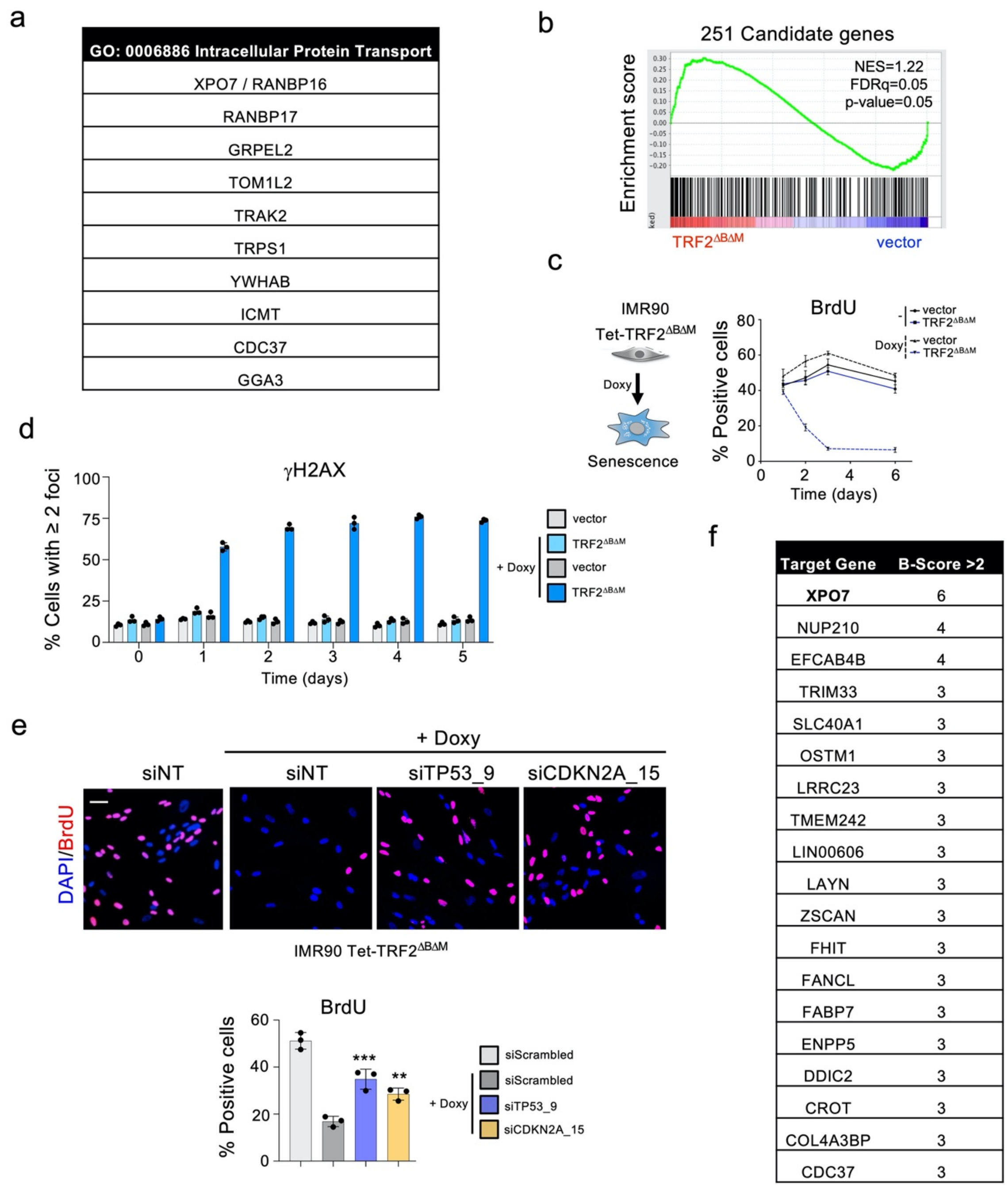

Supplemental Figure S2. Genetic screens identify XPO7 as a novel senescence regulator. (a) Candidate genes identified in the primary screen belonging to the GO term 'intracellular protein transport'. (b) GSEA showing the enrichment of a signature comprising the 251 candidate genes in the gene expression profile of IMR90 TRF2^{ΔBΔM} versus IMR90 vector cells. NES, Normalized enriched score; FDR, false discovery rate. (c) IMR90 Tet-TRF2^{ΔBΔM} cells. Treatment with 1000 ng/mL doxycycline induces TRF2^{ΔBΔM} and induces growth arrest, as assessed by measuring the percentage of cells incorporating BrdU. (d) Induction of TRF2^{ΔBΔM} causes DNA damage. Percentage

of cells with 2 or more γH2AX foci. (e) Knocking down *TP53* or *CDKN2A* (encoding for $p16^{INK4a}$) blunts the growth arrest caused by $TRF2^{\Delta B\Delta M}$ expression. Representative pictures (left) and quantification of the percentage of cells positive for BrdU (right) are shown. Scale bar represent 50 μm. (*n* = 3 independent experiments). ***P<0.001, **P<0.01. Statistical significance was calculated using one-way ANOVA with multiple comparison to siScrambled + doxy cells. All error bars represent mean ± s.d. (f) List of genes with at least 2 different siRNA sequences scoring with a BrdU B-score >2 in the secondary screen. The 'B-Score>2' column shows the number of individual data points for siRNAs with a B-Score>2 for each gene.

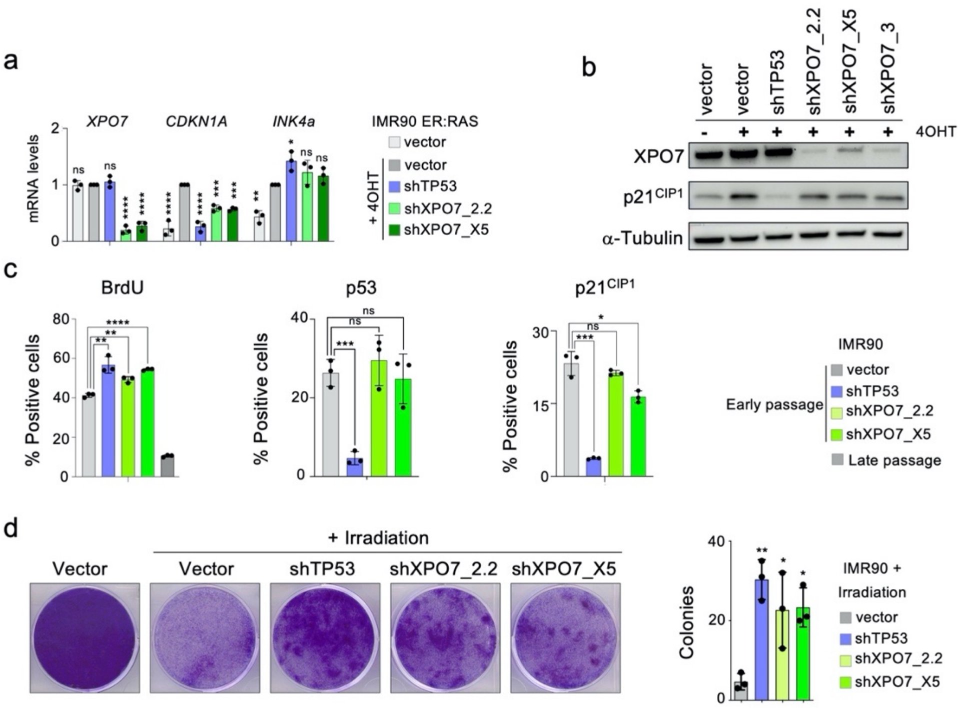

Supplemental Figure S3. Depletion of XPO7 expression with shRNAs. (a-b). IMR90 ER:Ras cells transduced with shRNA targeting TP53, XPO7 or a non-targeting control (vector) were treated with 4OHT where indicated to induce OIS. (a) The expression of *XPO7*, *CDKN1A* (encoding for $p21^{CIP1}$) and *INK4a* (encoding for $p16^{INK4a}$) was determined using RT–qPCR (n = 3 independent experiments). ****P<0.0001, ***P<0.001, **P<0.01, *P<0.05, n.s., non-significant. Statistical significance was calculated using one-way ANOVA with multiple comparison to control senescent cells. All error bars represent mean ± s.d. (b) Western blot showing XPO7 and $p21^{CIP1}$ expression. A representative western blot out of 3 is shown. (c) Effect of XPO7 knockdown in early passage IMR90 cells. Early passage IMR90 cells were transduced with shRNA targeting TP53, XPO7 or a non-targeting control (vector). BrdU incorporation (left), p53 (middle) and $p21^{CIP1}$ expression (right) were measured by IF. Late passage IMR90 cells were included as a reference. (n=3 independent experiments). Data represented as mean±SD. *P < 0.05; **P < 0.01; ***P < 0.001; ****P < 0.0001; ns, non-significant. Unpaired, two-tailed, Student's t-test. (d) IMR90 cells were transduced with shRNA targeting TP53, XPO7 or a non-targeting control (vector) and γ-irradiated (5Gy) where indicated. Representative pictures of crystal violet stained plates (left) and colony quantification (right) are shown. (n=3 independent experiments). Data represented as mean±SD. *P < 0.05; **P < 0.01. All comparisons refer to irradiated vector cells. Unpaired, two-tailed, Student's t-test.

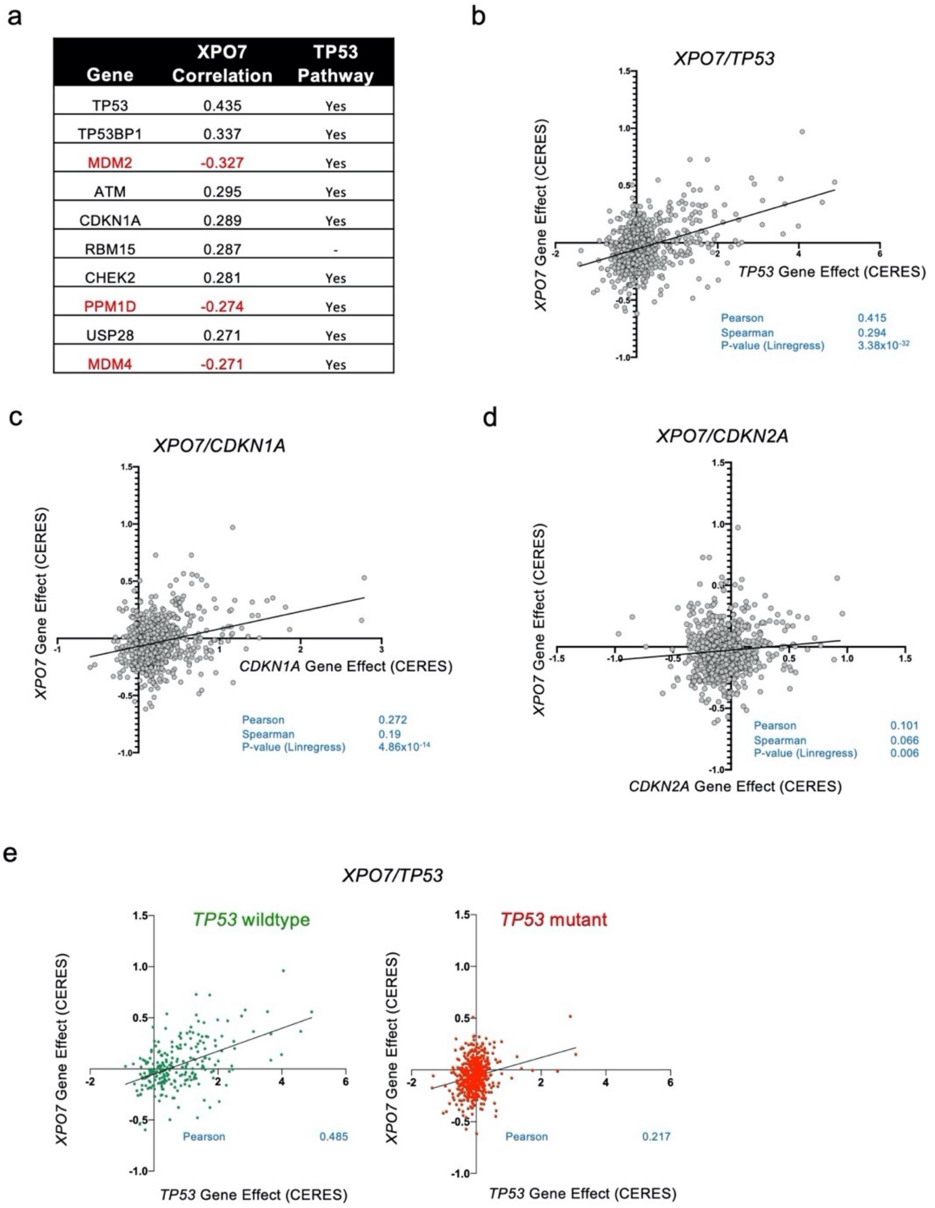

Gene	XPO7 Correlation	TP53 Pathway
TP53	0.435	Yes
TP53BP1	0.337	Yes
MDM2	-0.327	Yes
ATM	0.295	Yes
CDKN1A	0.289	Yes
RBM15	0.287	-
CHEK2	0.281	Yes
PPM1D	-0.274	Yes
USP28	0.271	Yes
MDM4	-0.271	Yes

Supplemental Figure S4. CRISPR screens suggest a functional correlation between XPO7 and the p53 pathway. (a) Table summarizing the top 10 genes for which the effect of knocking them out correlates with the effect of knocking out *XPO7* on the CRISPR AVANA dataset. 9/10 genes belong to the p53 pathway. The gene names are black for those that are positive regulators or effectors of the p53 pathway and in red for negative regulators. Correlation data is from depmap.org. (b-d) Correlation between the gene effect (CERES) on the CRISPR (Avana) Public 20Q1 dataset for *XPO7/TP53* (b), for *XPO7/CDKN1A* (c) and *XPO7/CDKN2A* (d). (e) Correlation between the gene effect (CERES) on the CRISPR (Avana) Public 20Q1 dataset for *XPO7/TP53.* Cell lines were classified according to their TP53 mutational status.

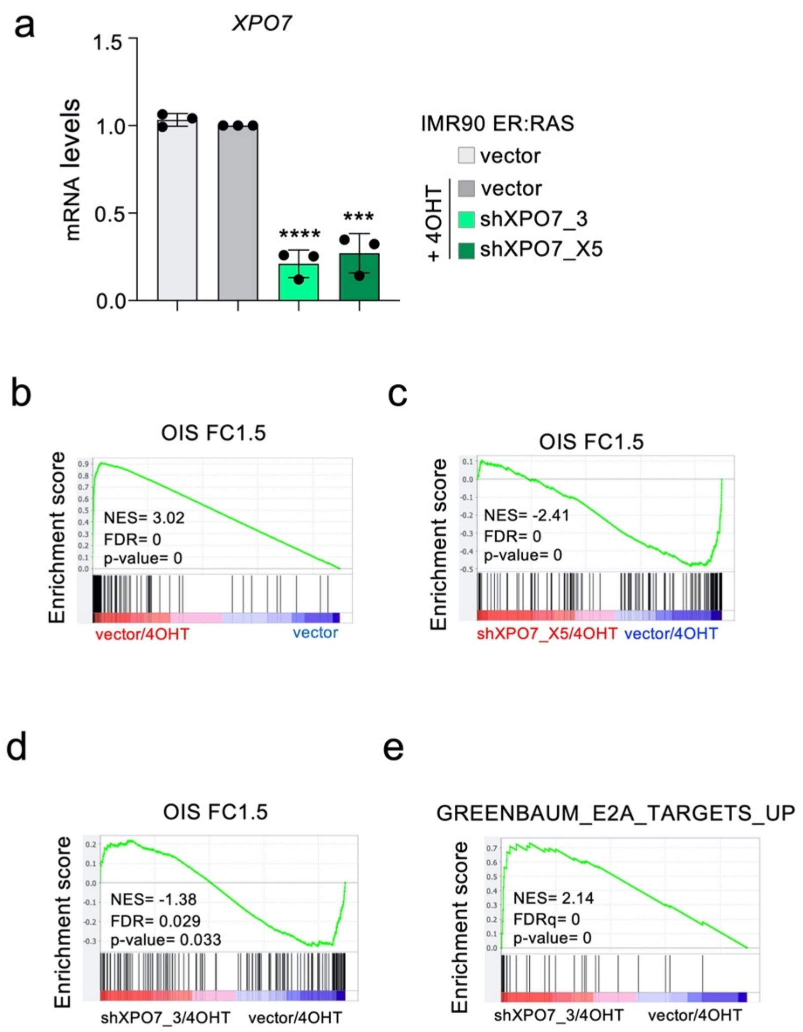

Supplemental Figure S5. XPO7 regulates TCF3 target genes during OIS. (a) The expression of *XPO7* was determined using RT–qPCR (*n* = 3 independent experiments). ****P<0.0001, ***P<0.001. Statistical significance was calculated using one-way ANOVA with multiple comparison to vector-infected senescent cells. All error bars represent mean ± s.d. (b-c) GSEA showing (b) enrichment of a signature associated with OIS (OIS FC1.5) during OIS (vector/4OHT versus vector) and (c) its depletion in cells undergoing senescence and infected with XPO7 shRNAs (shXPO7_X5/4OHT versus vector/4OHT).(d) GSEA showing the depletion of a signature associated with OIS (OIS FC1.5) on cells undergoing senescence and infected with XPO7 shRNAs (shXPO7_3/4OHT versus vector/4OHT). (e) GSEA showing the enrichment of a signature associated with the knockdown of TCF3 target genes (Greenbaum_E2A_targets_up) on cells undergoing senescence infected with XPO7 shRNAs (shXPO7_3/4OHT versus vector/4OHT). (NES) Normalized enriched score; (FDR) false discovery rate.

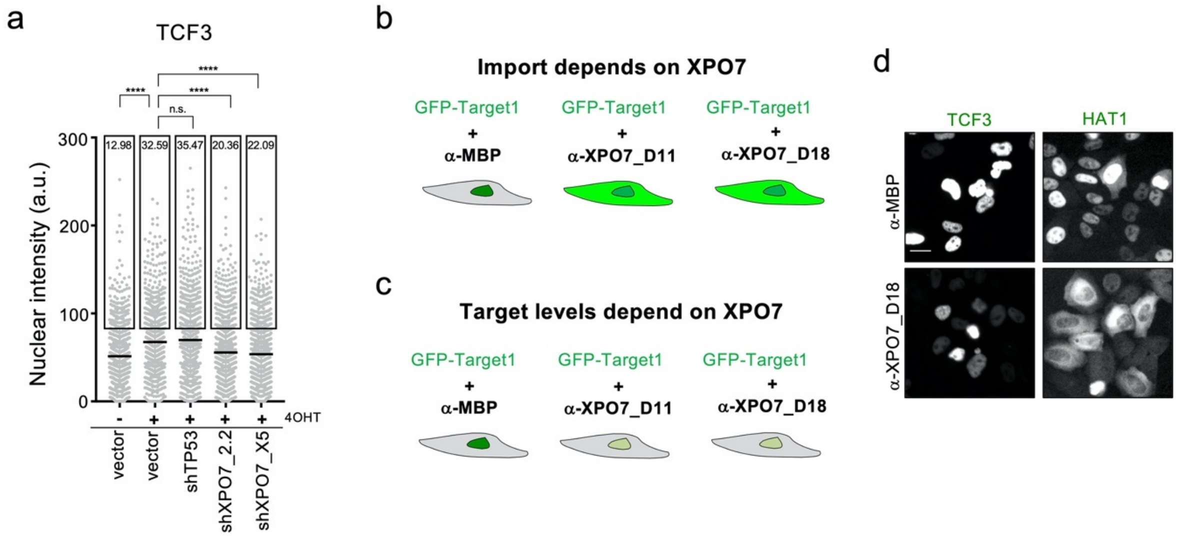

Supplemental Figure S6. XPO7 regulates TCF3 levels. (a) This data relates to Fig 5d. IMR90 ER:RAS cells were transduced with the indicated vectors and treated with 4OHT where indicated to induce OIS. TCF3 nuclear intensity values are shown. (n=1,000 cells per condition). A representative out of 3 experiments is shown. ****P<0.0001, n.s., non-significant. Statistical significance was calculated using one-way ANOVA with multiple comparison to control senescent cells. Values refer to the percentage of positive cells (those included in the boxes) and are equivalent to those used in Fig 5d. Bars indicate mean. (b-c) Scheme summarizing the nanobody system to validate the effect of XPO7 on potential targets. If XPO7 regulates the nuclear import of the target (b), we will observe changes in the nuclear/cytoplasmic ratio when co-transfecting nanobodies that target XPO7. If XPO7 regulates the overall levels of the target (c), a change in nuclear but no cytoplasmic signal will be detected. (d) Representative images of the experiment described in Fig. 5f. Scale bar, 20 μM.

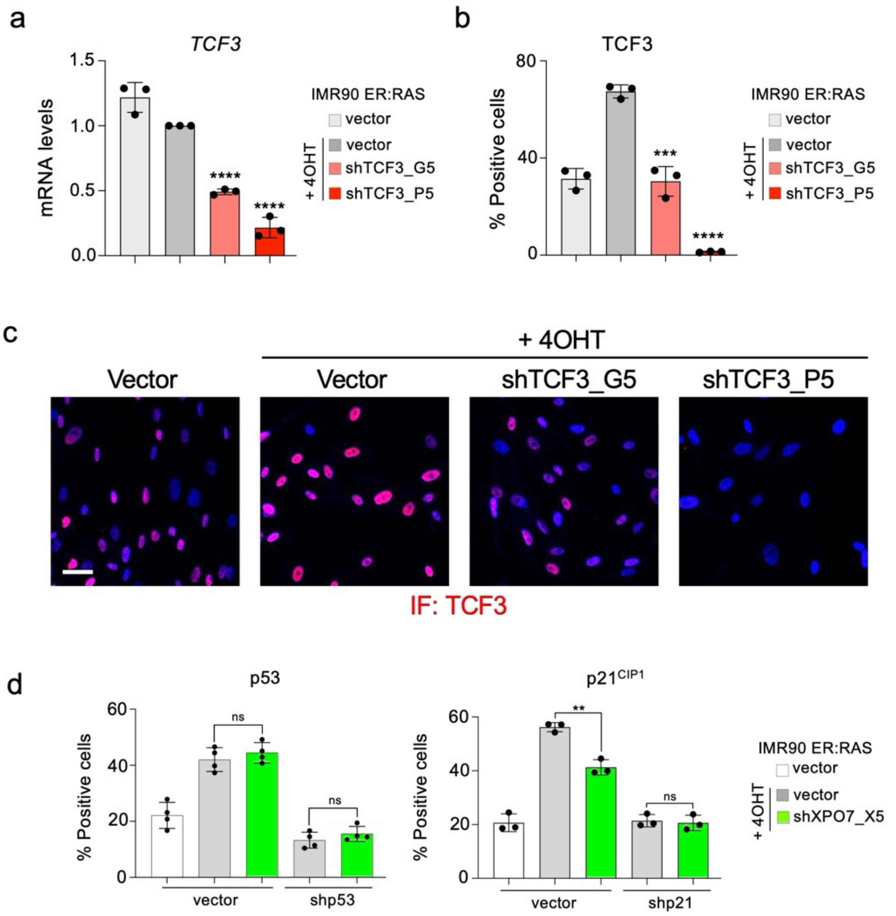

Supplemental Figure S7. Depletion of TCF3, p53 and p21^{CIP1} with shRNAs. (a-c) IMR90 ER:RAS cells transduced with shRNA targeting TCF3 or a non-targeting control (vector) were treated with 4OHT where indicated to induce OIS. (a) The expression of *TCF3* was determined using RT–qPCR. (n = 3 independent experiments). ****P<0.0001. Statistical significance was calculated using one-way ANOVA with multiple comparison to control senescent cells. All error bars represent mean ± s.d. (b-c) Levels of TCF3 were determined by quantitative IF. Representative pictures (b) and quantification (c) are shown. (n = 3 independent experiments). ****P<0.0001, ***P<0.001. Statistical significance was calculated using one-way ANOVA with multiple comparison to control senescent cells. All error bars represent mean ± s.d. Scale bar 50 µm. (d) Expression of p53 (left) or p21^{CIP1} (right) was evaluated by IF in the cells from the experiment described in Fig. 6c. (n = 3 independent experiments). **P<0.01; ns, non-significant. Statistical significance was calculated using unpaired, two-tailed, Student’s t-test. All error bars represent mean ± s.d.

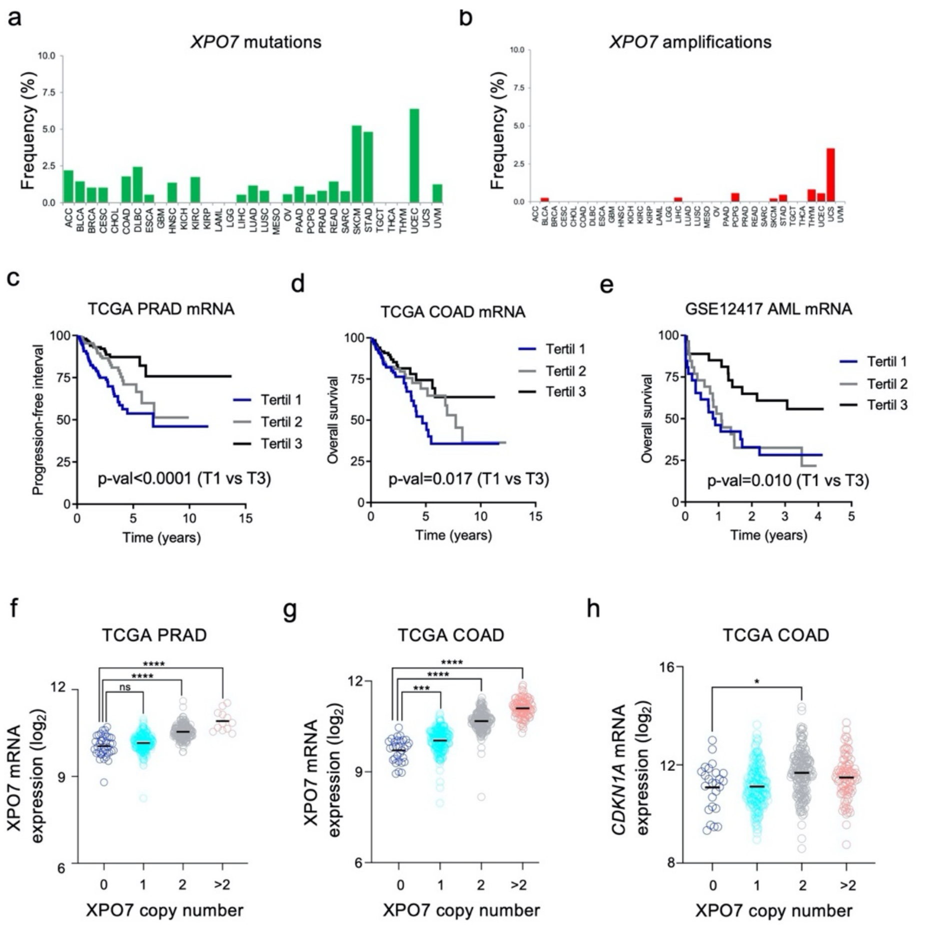

Supplemental Figure S8. Low *XPO7* expression is associated with worse cancer survival. (a-b) XPO7 alteration frequencies in 33 cancer types from the Pan-Cancer Atlas of the TCGA consortium. Data about mutations (a) and amplifications (b), are represented for each of the cancer types. Samples are ordered alphabetically from left to right. c-e) Survival curves from the TCGA prostate adenocarcinoma (c, PRAD) and colon adenocarcinoma (d, COAD) cohorts, and an acute myeloid leukemia (e, AML) cohort from (Metzeler et al. 2008) Tumors were ranked from lowest to highest mRNA values, and 3 groups were analyzed corresponding to 3 tertiles, from lowest (Tertile 1) to highest (Tertile 3) values. P-values were calculated using log-rank test and are shown for comparison between Tertile 1 (T1) and Tertile 3 (T3). Patients whose tumours display lower XPO7 expression have a worse prognosis. (f-g) Correlation between XPO7 copy number and XPO7 mRNA expression in samples from the TCGA PRAD (f) and COAD (g) cohorts. (h) Correlation between XPO7 copy number and CDKN1A mRNA expression in samples from the TCGA COAD cohort. XPO7 copy number: 0, deep deletion; 1, shallow deletion; 2, wildtype; >2, amplifications. P-values for f-h were calculated using one-way ANOVA with Dunnett's T3 multiple comparisons tests.

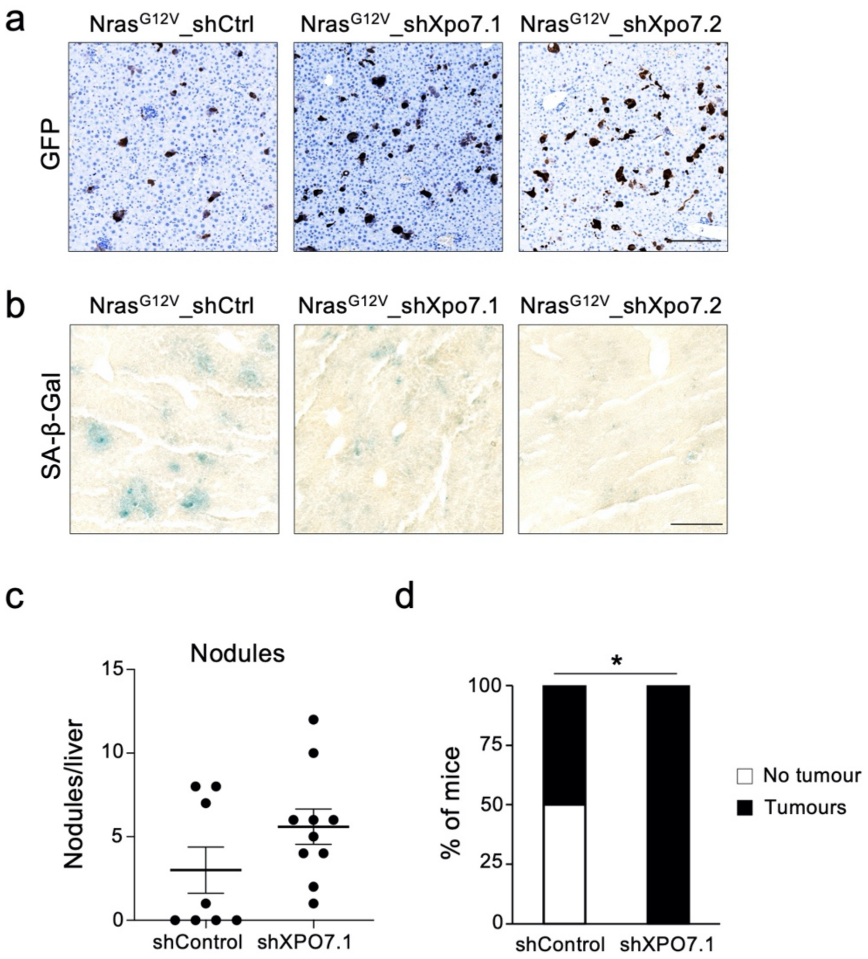

Supplemental Figure S9. Effect of Xpo7 deletion in OIS and liver cancer. (a-b) Related to Fig. 7f-g. Representative images of GFP immunohistochemistry (a) and SA-β-gal staining (b) in liver sections. Scale bar, 50 µm. (c-d) Related to Fig. 7i-k. Liver nodules (c) and percentage of mice with tumours (d). (n=8 mice for shCtrl and n=10 mice for shXpo7.1). Statistical significance was calculated using Fisher exact test of the tumour vs non-tumour mice in the shControl vs shXPO7.1 cohorts.

Supplemental Table S1. Plasmids used in this study.

Plasmid	Origin	Identifier
pWZL Hygro TRF2$^{\Delta B\Delta M}$	Addgene	18013
pBABE-puro	Addgene	1764
pBABE-puro-TRF2$^{\Delta B\Delta M}$	This paper	-
pBABE-puro-Ras^{G12V}	Addgene	1768
pLenti-CMVtight eGFP Puro	Addgene	w771-1
pLenti-CMV rtTA3	Addgene	w785-1
pEGFP-TCF3	This paper	-
tet-Vector	This paper	-
tet-TRF2$^{\Delta B\Delta M}$	This paper	-
tet-TCF3	This paper	-
pCaNIGmirE-5'-RAS^{G12V}-shXpo7_1	This paper	-
pCaNIGmirE-5'-RAS^{G12V}-shXpo7_2	This paper	-
pRRL-miRE	(Fellmann et al. 2011)	-
pGIPZ	Thermo Fisher Scientific/ LMS Genomic Facility	-

Supplemental Table S2. shRNAs used in this study.

shRNA*	Sense sequence
shTP53	GGAGGATTTCATCTCTTGTAT
shp21	ATCTGGCATTAGAATTATTTAA
shXPO7_2.2	AGGATGACTATGTCTTCAG
shXPO7_X3	CAGGCTTCAGGAAAGAATCTAA
shXPO7_X5	GTGCGGTACAGTTCATGCTGAA
shTCF3_G5	CGGGCACATGTGAAAGGTA
shTCF3_P5	TATGTTTCCATTTCTCCGCTTT
shXpo7_1	CCAGCAAGATGATAACAATGTA
shXpo7_2	TCCGGAACTATGTGCTCAACTA

*All shRNAs target the corresponding human genes, except shXpo7_1 and shXpo7_2 that target the mouse *Xpo7* gene.

Supplemental Table S3. siRNAs used in this study.

siRNA*	Sense sequence
Hs_CDKN2A_15	TACCGTAAATGTCCATTTATA
Hs_TP53_3	CAGAGTGCATTGTGAGGGTTA
Hs_XPO7_5	CAAGCTTGTATCACGCACAAA
Hs_XPO7_6	AAGGCTGACATGGCTGGTTTA

*All siRNAs target the corresponding human genes.

Supplemental Table S4. Antibodies used in this study.

Antibody	Clone	Species	Application
BrdU (BD Pharmigen, 555627)	3D4	Mouse	IF
BrdU (Abcam, ab6326)	BU1/75 (ICR1)	Rat	IF
p16^{INK4a} (Santa Cruz, sc-56330)	JC8	Mouse	IF
p21^{Cip1} (Cell Signalling, 2947S)	12D1	Rabbit	IF
p21^{Cip1} (Santa Cruz, sc-471)	M19	Rabbit	IF
p53 (Santa Cruz, sc-126)	DO-1	Mouse	IF
53BP1 (Novus, NB100-304)	Polyclonal	Rabbit	IF
γH2AX (Millipore, 05-636)	JBW301	Mouse	IF
TCF3 (Sigma Aldrich, HPA062476)	Polyclonal	Rabbit	IF
GFP (Abcam, EPR14104)	EPR14104	Rabbit	IHC
GFP (Abcam, ab13970)	Polyclonal	Chicken	IHC
Ki67 (Abcam, ab16667).	SP6	Rabbit	IHC
TRF2 (Abcam ab13579)	4A794	Mouse	IF, WB
α-Tubulin (Sigma Aldrich, T6074)	B-5-1-2	Mouse	WB
α-Tubulin (Santa Cruz, sc-8035)	TU-02	Mouse	WB
α-Tubulin (Cell Signalling, 3873)	DM1A	Mouse	WB
Histone H3 (abcam, ab1791)	Polyclonal	Rabbit	WB
XPO7 (Santa Cruz, sc-390025)	A11	Mouse	WB
Anti-Mouse IgG AlexaFluor® 488 (Invitrogen, A11029)	Polyclonal	Goat	IF
Anti-Mouse IgG AlexaFluor® 594 (Invitrogen, A11032)	Polyclonal	Goat	IF
Anti-Rabbit IgG AlexaFluor® 594 (Invitrogen, A11037)	Polyclonal	Goat	IF
Anti-Rabbit IgG AlexaFluor® 488 (Invitrogen, A21441)	Polyclonal	Chicken	IF
Anti-Rat IgG AlexaFluor® 488 (Invitrogen, A11006)	Polyclonal	Goat	IF
Anti-Mouse IgG HRP (Santa Cruz, sc-2005	Polyclonal	Goat	WB
Anti-Chicken IgG AlexaFluor® 488 (Invitrogen, A11039)	Polyclonal	Goat	IF
Anti-Mouse IgG AlexaFluor® 488 (Invitrogen, A11001)	Polyclonal	Goat	IF

*IF, immunofluorescence; IHC, immunohistochemistry; WB, Western blot

Supplemental Table S5. Primers used in this study.

Name	Sequence
human CDKN1A F	CCTGTCACTGTCTTGTACCCT
human CDKN1A R	GCGTTTGGAGTGGTAGAAATC
human INK4A F	CGGTCGGAGGCCGATCCAG
human INK4A R	GCGCCGTGGAGCAGCAGCAGCT
human RPS14 F	CTGCGAGTGCTGTCAGAGG
human RPS14 R	TCACCGCCCTACACATCAAACT
human XPO7 F	CTTGAAGTACTGGGGCCGTT
human XPO7 R	GCTCGCTCGTGTGATTGTTC
human TCF3 F	GGACTCGGAGGCAAGAGC
human TCF3 R	AGTACTGGGAGGTCCCCTTC
mouse XPO7 R	GAATGGAGCAAAATGGCGGA
mouse XPO7 F	CAGCTGGGAGTAGGACGAACTT
mouse GAPDH F	AACTTTGGCATTGTGGAAGG
mouse GAPDH R	ACACATTGGGGGTAGGAACA

SUPPLEMENTAL MATERIALS AND METHODS

Cell lines and culture. HEK-293T, HeLa and IMR90 cells were obtained from ATCC. To generate IMR90 cells expressing TRF2$^{\Delta B\Delta M}$, ER:RAS^{G12V}, miR30-based shRNAs (pGIPz) and miRE-based shRNA (pRRL) retroviral and lentiviral infections were carried out as previously described (Banito et al. 2009; Aarts et al. 2017). To generate IMR90-tet-control, IMR90-tet-TRF2$^{\Delta B\Delta M}$, and IMR90-tet-TCF3 cells, IMR90 cell were infected with equal amounts of lentivirus containing rtTA3, and the iCMV-tight vector expressing the relevant cDNA (linker DNA, TRF2$^{\Delta B\Delta M}$, or E47 cDNA respectively). To select cells that efficiently integrated both constructs the cells were treated with 0.5 mg/ml Puromycin and 25 mg/ml Hygromycin. Cells were cultured in D-MEM supplemented with 10% foetal calf serum (tetracycline free in the case of tet-inducible vectors) and 1% Antibiotic-antimycotic (Gibco 15240) grown at 37°C in 5% CO_2. Unless specified, cells were cultured until sub-confluence then passaged and split 1 in 4. To induce OIS, IMR90 ER:RAS cells were treated with 100 nM 4-hydroxytamoxifen (4OHT; Sigma) reconstituted in DMSO, and to induce TRF2$^{\Delta B\Delta M}$ or TCF3 expression, tet-inducible cells were treated with 100 or 1000ng/ml (as specified) of doxycycline (dox; Sigma).

Primary shRNA screen. For the initial screen, we used a pGIPZ human genome-wide shRNA library consisting of ~58,000 lentiviral constructs. The library was divided into 12 pools, packaged into lentiviruses and introduced into TRF2$^{\Delta B\Delta M}$,-infected IMR90 fibroblasts in triplicate at a multiplicity of ~0.3. Samples from screening pools were collected three days post-infection (reference, day 0) and then at regular intervals over a 42-day culture period. Two pools (pool 1 and 10) were sequenced at baseline (day 0) and 5 serial time points (Day 13, 20, 27, 35 and 42) and shRNA representation was tracked over time. Preliminary analysis revealed that fold change in shRNA copies between day 0 and day 27 provided the optimal window to determine enrichment whilst minimising bias, therefore all remaining pools were sequences at day 0 and day 27 only. Library preparation, processing and analysis for determining shRNA enrichment was carried out as described before (Aarts et al. 2017). Following filtering for adequate representation at day 0, and significant fold enrichment at day 27, 251 candidates were shortlisted.

Secondary siRNA screen. A Flexiplate™ custom library targeting 185 of the 251 candidates with 4 individual siRNA per genes was obtained from Qiagen (limited by siRNA availability). For the screen, iCMV-TRF2$^{\Delta B \Delta M}$ cells were reverse transfected with siRNA that had been pre-dispensed into flat bottom 96 well tissue culture plates. Eighteen hours following transfection the media was replenished and supplemented with doxycycline (100 ng/mL) to induce the expression of TRF2$^{\Delta B \Delta M}$ and trigger senescence. Proliferation was then assessed after 3 days by measuring BrdU incorporation by immunofluorescence using the high throughput microscope (InCell 2000). B-score normalization was performed using the online Web cellHTS2 tool (http://web-cellhts2.dkfz.de/cellHTS-java/cellHTS2) (Pelz et al. 2010).

High Content Analysis. IF imaging was carried out using the automated high-throughput fluorescent microscope IN Cell Analyzer 2000 (GE Healthcare) with a 20x objective with the exception of DNA damage foci analysis which required a 40x objective. Fluorescent images were acquired for each of the fluorophores using built-in wavelength settings ('DAPI' for DAPI, 'FITC' for AlexaFluor® 488 and 'Texas Red' for AlexaFluor® 594). Multiple fields within a well were acquired in order to include a minimum of 1,000 cells per sample-well. HCA of the images were processed using the INCell Investigator 2.7.3 software as described previously (Herranz et al. 2015). Briefly, DAPI served as a nuclear mask hence allowing for segmentation of cells with a Top-Hat method. Nuclear IF in the reference wavelength, i.e. all wavelengths other than DAPI, were quantified as an average of pixel intensity (grey scale) within the specified nuclear area. The percentage positive cells were then determined using a threshold value determined from the control cell condition as the baseline. For DNA damage foci, nuclear foci were identified using the organelle function and nuclear foci were quantified as n number of foci per nucleus. Once thresholds had been determined, all image quantification was automated.

Immunofluorescence and FISH. Slides were incubated with blocking solution (10% goat serum in PBS1X) for 30 min at 37°C and stained with mouse-anti-γH2AX (1/500, Millipore 05-636) for 1 hour at 37°C followed by O/N incubation at 4°C. After three washes in PBS1X, slides were incubated with secondary goat anti-mouse Alexa 488 antibody (1/400, Life Technologies A11001) for 40 min at 37°C, washed three times

in PBS1X, post fixed for 10 min and incubated with ethanol series (70%, 80%, 90%, 100%). Slides were hybridized for 3 min at 80°C on a heat block with a solution containing a Cy3-O-O-(CCCTAA)3 probe (PNA bio) in 70% formamide, 10 mM Tris pH 7.4 and 1% blocking reagent (Roche, 11096176001). After 2 hours hybridisation at RT, slides were washed twice 15 min in 70% formamide, 20 mM Tris pH 7.4, followed by three washes of 5min each in 50 mM Tris pH 7.4, 150 mM NaCl, 0.05% Tween-20, dehydrated followed in successive ethanol baths and slides were air-dried. Slides were mounted with antifade reagent (ProLong Gold, Invitrogen) containing DAPI and images were captured with Zeiss microscope using Carl Zeiss software.

Microarray and RNA-Sequencing. For microarray experiments, cDNA was hybridized to Human Gene 1.0 ST arrays (Affymetrix) following manufacturer's instructions. Three biological replicates were performed for each condition. Microarray data processing and analysis was carried out at EMBL-GeneCore (Heidelberg, Germany). Microarray data was normalized using Robust Multichip Average (RMA) method available in "oligo" Bioconductor package and significant differentially expressed probesets were identified using Limma (Ritchie et al. 2015) with Benjamini-Hochberg corrected p-value < 0.05.

RNA-Seq libraries were prepared from 500 ng of total RNA using the Illumina Truseq mRNA stranded library prep kit (Illumina Inc. San Diego, USA) according to the manufacturer's protocol. Library quality was checked on a Bioanalyser HS DNA chip and concentrations were estimated by Qubit measurement. Libraries were pooled in equimolar quantities and sequenced on a Hiseq2500 using single end 50 bp reads. At least 20 million reads passing filter were achieved per sample. Sequencing reads from the RNA-Seq experiments were aligned to hg19 genome using Tophat v2.0.11 (Trapnell et al. 2009) using parameters "--library-type fr-firststrand --b2-very-sensitive --b2-L 25" and using known transcripts annotation from ensembl gene v72. Number of reads counts on exons were summarised using HTSeq v0.5.3p9 with "--stranded=reverse" option and differentially expressed genes were identified using DESeq2 (Love et al. 2014). Genes were ranked by fold change and Gene Set Enrichment Analysis was performed using GSEA v2.07 (Broad Institute) pre-ranked module.

Immunohistochemistry of liver sections. Liver samples were fixed in 4% PFA overnight before embedding and sectioning. Paraffin-embedded sections were rehydrated with a series of Histoclear then 100%, 75%, 50% and 25% ethanol and distilled water. Citrate buffer (pH 6.0) was used for antigen retrieval. The sections were then blocked (Dako) and incubated overnight with the following primary antibodies: rabbit anti-GFP (Abcam, EPR14104), chicken anti-GFP (Abcam, ab13970) and rabbit anti-Ki67 (Abcam, ab16667). SignalStain® Boost IHC Detection Reagent and SignalStain® DAB Substrate Kit (Cell Signaling) were used for DAB staining. Alexa Fluor conjugated secondary antibodies (Life technologies) were used for immunofluorescence. All images were acquired with ZEISS Axio Scan.Z1 at 20x magnification and then quantified with QuPath software. In particular, to identify Ki67+ cells, mean Ki67 nuclear intensity + standard deviation was used as the threshold for each image to control the staining variations.

SA-β-gal staining for tissue sections. Frozen sections were washed in cold PBS, fixed in cold 0.5% glutaraldehyde for 10 min, washed in 1mM $MgCl_2$/PBS (pH 6.0) and then incubated with X-gal staining solution for 16 hr at 37°C. After washing, sections were dehydrated in ethanol and Histoclear and mounted for image acquisition with ZEISS Axio Scan.Z1 at 20x magnification. ImageJ was used for quantification and SA-β-gal index was calculated as multiplying the percentage of positive areas and mean intensity value, as previously published (Tordella et al. 2016).

SUPPLEMENTAL REFERENCES

Aarts M, Georgilis A, Beniazza M, Beolchi P, Banito A, Carroll T, Kulisic M, Kaemena DF, Dharmalingam G, Martin N et al. 2017. Coupling shRNA screens with single-cell RNA-seq identifies a dual role for mTOR in reprogramming-induced senescence. *Genes Dev* **31**: 2085-2098.

Banito A, Rashid ST, Acosta JC, Li S, Pereira CF, Geti I, Pinho S, Silva JC, Azuara V, Walsh M et al. 2009. Senescence impairs successful reprogramming to pluripotent stem cells. *Genes Dev* **23**: 2134-2139.

Fellmann C, Zuber J, McJunkin K, Chang K, Malone CD, Dickins RA, Xu Q, Hengartner MO, Elledge SJ, Hannon GJ et al. 2011. Functional identification of optimized RNAi triggers using a massively parallel sensor assay. *Mol Cell* **41**: 733-746.

Herranz N, Gallage S, Mellone M, Wuestefeld T, Klotz S, Hanley CJ, Raguz S, Acosta JC, Innes AJ, Banito A et al. 2015. mTOR regulates MAPKAPK2 translation to control the senescence-associated secretory phenotype. *Nat Cell Biol* **17**: 1205-1217.

Love MI, Huber W, Anders S. 2014. Moderated estimation of fold change and dispersion for RNA-seq data with DESeq2. *Genome Biol* **15**: 550.

Metzeler KH, Hummel M, Bloomfield CD, Spiekermann K, Braess J, Sauerland MC, Heinecke A, Radmacher M, Marcucci G, Whitman SP et al. 2008. An 86-probe-set gene-expression signature predicts survival in cytogenetically normal acute myeloid leukemia. *Blood* **112**: 4193-4201.

Pelz O, Gilsdorf M, Boutros M. 2010. web cellHTS2: a web-application for the analysis of high-throughput screening data. *BMC Bioinformatics* **11**: 185.

Ritchie ME, Phipson B, Wu D, Hu Y, Law CW, Shi W, Smyth GK. 2015. limma powers differential expression analyses for RNA-sequencing and microarray studies. *Nucleic Acids Res* **43**: e47.

Tordella L, Khan S, Hohmeyer A, Banito A, Klotz S, Raguz S, Martin N, Dhamarlingam G, Carroll T, Gonzalez Meljem JM et al. 2016. SWI/SNF regulates a transcriptional program that induces senescence to prevent liver cancer. *Genes Dev* **30**: 2187-2198.

Trapnell C, Pachter L, Salzberg SL. 2009. TopHat: discovering splice junctions with RNA-Seq. *Bioinformatics* **25**: 1105-1111.
